# Anacardium Occidentale L. Leaf Extracts Protect Against Glutamate/H_2_O_2_-Induced Oxidative Toxicity and Induce Neurite Outgrowth: The Involvement of SIRT1/Nrf2 Signaling Pathway and Teneurin 4 Transmembrane Protein

**DOI:** 10.3389/fphar.2021.627738

**Published:** 2021-04-23

**Authors:** Chatrawee Duangjan, Panthakarn Rangsinth, Shaoxiong Zhang, Michael Wink, Tewin Tencomnao

**Affiliations:** ^1^ Graduate Program in Clinical Biochemistry and Molecular Medicine, Department of Clinical Chemistry, Faculty of Allied Health Sciences, Chulalongkorn University, Bangkok, Thailand; ^2^ Department of Clinical Chemistry, Faculty of Allied Health Sciences, Chulalongkorn University, Bangkok, Thailand; ^3^ Leonard Davis School of Gerontology, University of Southern California, Los Angeles, CA, United States; ^4^ College of Horticulture, Fujian Agriculture and Forestry University, Fuzhou, China; ^5^ Institute of Pharmacy and Molecular Biotechnology, Im Neuenheimer Feld 364, Heidelberg University, Heidelberg, Germany; ^6^ Natural Products for Neuroprotection and Anti-Ageing Research Unit, Department of Clinical Chemistry, Faculty of Allied Health Sciences, Chulalongkorn University, Bangkok, Thailand

**Keywords:** glutamate, H_2_O_2_, neurite outgrowth, teneurin-4, Nrf2/SIRT1, anacardium occidentale

## Abstract

Neurodegenerative diseases are linked to neuronal cell death and neurite outgrowth impairment that are often caused by oxidative stress. Natural products, which have neuroprotective against oxidative stress and neurite outgrowth inducing activity, could be potential candidates for alternative treatment of neurodegenerative diseases. This study aims to investigate the neuroprotective effects and neuritogenesis properties of *Anacardium occidentale* leaf extracts in cultured neuronal (HT22 and Neuro-2a) cells. We found gallic acid, catechin and quercetin as the main compounds in *A. occidentale* extracts. The extracts have a protective effect against glutamate/H_2_O_2_-mediated oxidative stress-induced cell toxicity. The gene expression of cellular antioxidant enzymes (SODs, GPx and, GSTs) were up-regulated by this treatment. The treatment also triggered SIRT, Nrf2 proteins as well as the mRNA transcriptions of relevant anti-oxidation genes (NQO1, GCLM, and EAAT3). We demonstrated that the extracts promote antioxidant defense in neuronal cells via the SIRT1/Nrf2 signaling pathway. Moreover, the extracts increase neurite outgrowth and Ten-4 expression in Neuro-2a cells. However, the neuritogenesis properties did not occur, when Ten-4 expression was knocked down by corresponding siRNA. These results suggest that the leaf extracts have an interesting neuritogenesis and neuroprotective potential against glutamate/H_2_O_2_-mediated toxicity and could be a potential therapeutic candidate for neurodegenerative diseases.

## Introduction

Neurogenesis describes the process of growth, survival, proliferation, differentiation and regeneration of neurons (Lam et al., 2016). Impairment of neurogenesis affects neuronal differentiation and neuronal cell loss in various neurodegenerative disorders (Lam et al., 2016). During neuronal differentiation, neurite outgrowth is an essential step for functional networks (connectome) of neurons. Regulation of neurite outgrowth can promote neuronal regeneration from nerve injury or neurological disorders, which plays an important role in development of therapies for neurodegenerative diseases (Bonaterra et al., 2018). Teneurin-4 (Ten-4), a transmembrane protein, is highly expressed in the central nervous system and plays a role in neurogenesis. Ten-4 expression mediates neurite outgrowth of the Neuro-2a cells (Suzuki et al., 2014).

Glutamate, the main excitatory neurotransmitter in the brain, has been recognized as one initiating factor for several neurodegenerative disorders (Jin et al., 2014; Sukprasansap et al., 2017). High levels of glutamate activate structural degradation, ROS/RNS production, mitochondrial and DNA damage, which further lead to neurotoxicity and neuronal cell damage (Jin et al., 2014; Hirata et al., 2018). Glutamate oxidative stress and neurotoxicity play a major role in a variety of neurodegenerative diseases, especially Alzheimer’s disease (AD) (Jin et al., 2014; Mittal et al., 2014). Sirtuin 1 (SIRT1) is a Class III histone deacetylases that plays an important role in physiological and biochemical cell processes, including aging, inflammation and neuroprotection (Xue et al., 2016). SIRT1 regulates transcription factors, including nuclear factor-E2-related factor 2 (Nrf2) that is a major regulator in antioxidant defenses. Evidence suggests that SIRT1 and Nrf2 are involved in the CNS redox balance of neurodegenerative disorders by promoting antioxidant responses (Peng et al., 2017; Bi et al., 2018). In addition, enhancing SIRT1 and Nrf-2/HO-1 expression can protect neurons against oxidative injury in neuronal cells (Peng et al., 2017; Bi et al., 2018).

Reduction of oxidative stress and induction of neuronal differentiation are key parameters for neuroprotective effects. Thus, natural products from herbs or plant extracts with antioxidative and neuroprotective properties could provide an alternative approach to treat neurodegenerative diseases.

*Anacardium occidentale* L (AO) originates from Brazil, but is presently cultivated in many tropical countries around the globe. Leaf extracts from *A. occidentale* have been widely used as food and medicine in Thailand. The secondary metabolites which are presented in *Anacardium* plants exhibit substantial anti-oxidant (Salehi et al., 2019; Fusco et al., 2020; Siracusa et al., 2020), anti-inflammatory (Fusco et al., 2020; Siracusa et al., 2020) and anti-microbial (Muroi and Kubo, 1996; Salehi et al., 2019) effects. A recent study reported high content of antioxidant bioactive secondary metabolites from *A. occidentale* leaf extracts, including quercetin 3-O-glucoside, quercetin 3-(2-galloylglucoside), quercetin 3-arabinoside, quercitrin/kaempferol-7-O-glucoside, α-tocopherols, and salicylic acid (Duangjan et al., 2019a). Moreover, the leaf extracts exerted anti-aging and oxidative-stress resistance in the C*aenorhabditis elegans* model (Duangjan et al., 2019a). However, neuritogenesis and neuroprotective effects of *A. occidentale* leaf extracts against oxidative stress in neuronal cell models have not been reported.

In the current study, the effects of leaf extracts from *A. occidentale* for neuroprotection against glutamate/H_2_O_2_-induced cytotoxicity and neuroregeneration in cultured neuronal (HT22 and Neuro-2a) cells were investigated. This study provides the first evidence that these leaf extracts induce neurite outgrowth and exert a neuroprotective effect via the SIRT1/Nrf2 signaling pathway.

## Materials and Methods

### Chemicals and Reagents

H_2_DCFDA were obtained from Molecular Probes (Eugene, OR, United States), MTT from Bio Basic (Markham, Ontario, Canada), DMSO, DMEM and FBS from Sigma-Aldrich (St. Louis, MO, United States), The RIPA buffer was purchased from Abcam (Cambridge, United Kingdom), Trizol from Invitrogen (Carlsbad, CA, United States), and the penicillin/streptomycin solution from Gibco (Waltham, MA, United States). The CytoTox 96^®^ kit for LDH assay obtained from Promega (Madison, WI, United States), RT PreMix and qPCR Master Mix solution from Bioneer (Daejeon, South Korea).

Primary antibodies for western blot analysis sirtuin 1 (SIRT1) (RRID:AB_1196631), nuclear factor-E2-related factor 2 (Nrf2) (RRID:AB_1950359), and β-actin (RRID:AB_2750839) antibodies were purchased from Cell Signaling Technology (Danvers, MA, United States), Teneurin-4 (RRID:AB_10920937) antibody from R&D Systems, Inc (MN, Canada) and GAP43 (RRID:AB_598153) antibody from Abcam (Cambridge, United Kingdom). Secondary (RRID: AB_2099233, RRID: AB_330924) antibodies were purchased from Cell Signaling Technology (Danvers, MA, United States).

### Plant Extraction

The leaves of *Anacardium occidentale* L (AO) were collected from Jana district, Songkhla Province, in southern Thailand (7.205278° N, 100.596944° E) by Mrs. Laong Kwunpet and Mrs. Korakod Choosri. The leaves were stored as a voucher specimen (No. BCU-015863) at the herbarium of Kasin Suvatabhandhu (Department of Botany, Faculty of Science, Chulalongkorn University, Thailand). The leaves were sequentially extracted with hexane, dichloromethane and methanol using a Soxhlet apparatus as described before (Duangjan et al., 2019a).

### Qualitative Phytochemical Screening

The secondary metabolites of the hexane and methanol extract were submitted to characterize and quantify the bioactive compounds by Gas/Liquid Chromatography-Mass Spectrometry (GLC-MS) and High-Performance Liquid Chromatography (HPLC) (Duangjan et al., 2019b) at the RSU Science and Technology Research Equipment Center (Rangsit University, Thailand) (Supplementary Materials).

### Cell Culture

Mouse hippocampal neuronal HT22 cells have been used to study the neuroprotective properties. These cells lack ionotropic glutamate receptors and are resistant to excitotoxicity as a cause for glutamate-stimulated neuronal death (Sukprasansap et al., 2017). Mouse neuroblastoma Neuro-2a cells have been extensively used to study neuronal differentiation and neurite growth (Park et al., 2015). Thus, HT22 cells and Neuro-2a cells were used for the neuronal cell models in this study.

HT22 cells (Salk Institute, San Diego, CA, United States) and Neuro-2a cells (The JCRB Cell Bank, JCRB No. IF050081, Lot No. 09262014) were cultured in DMEM (HT22) or, DMEM and HamF12 (Neuro-2a), supplemented with 10% (v/v) FBS and 1% streptomycin followed under 5% CO_2_, 37°C condition.

### Cell Treatment

HT22 and Neuro-2a cells were pretreated with different concentrations of *A. occidentale* hexane extract (AOH) (10–50 μg/ml) and *A. occidentale* methanol extract (AOM) (0.5–10 μg/ml) for 48 h. To induce cell toxicity, glutamate or H_2_O_2_ were added to the culture medium. For protective assays, the extracts were co-treatment with glutamate or H_2_O_2_ for 18–24 h or 15 min respectively. Stock solutions of glutamate and H_2_O_2_ were prepared in DMEM. Stock solutions of the AO extracts were prepared in DMSO. For the untreated control group, cells were treated with 0.1% (v/v) DMSO. For the positive control group, cells were treated with 4 μM quercetin.

### Determination of Cell Viability

Cell viability was evaluated by using MTT and LDH assays (Supplementary Materials).

### Measurement of Intracellular Reactive Oxygen Species

ROS production was quantified by the DCFH-DA method. After treatment, 10 μM H2DCFDA was added to the culture medium and incubated for 30 min at 37°C, followed by washing with Hank’s balanced salt solution (HBSS). Fluorescence intensity (excitation = 485 nm; emission = 535 nm) was measured using an EnSpire^®^ Multimode Plate Reader (Perkin-Elmer). Data were expressed as the percentage of fluorescence intensity of treated cells relative to the untreated control.

### RNA Isolation and Quantitative RT-PCR

Total RNA was extracted using a trizol reagent (Invitrogen) following the manufacturer’s instructions. All real-time PCR reactions were performed in an Exicycler™ 96 (Bioneer). The gene-specific sequences of the primers were SOD1, CAT, GPx, GSTo1, GSTa2, NQO1, GCLM, EAAT3 and β-actin as normalization control (Prasansuklab et al., 2017; Sukprasansap et al., 2017) (Supplementary Materials).

### Western Blot Analysis

Whole-cell lysates were prepared in 1× RIPA buffer for 45 min on ice according to the manufacturer’s protocol. The cell lysates were collected and the protein concentration was determined using the Bradford protein assay. An equal amount of protein (20 μg) was denatured by heating in Laemmli loading buffer at 95°C for 10 min, subsequently separated on 6–10% SDS polyacrylamide gel and then transferred to PVDF membranes.

After blocking for 2 h with 5% skimmed milk in tris buffered saline (TBS-T, 0.1% Tween 20), the membranes were allowed to incubate overnight at 4°C with primary antibodies specific for SIRT1 (1:2000), Nrf2 (1:8000), GAP43 (1:8000), Ten-4 (1:2000) or β-actin (1:16,000). Membranes were washed three times with TBST for 15 min and incubated with HRP-conjugated secondary antibodies (1:10,000) at room temperature for 60 min. Subsequently, the bands were visualized using a film exposure with the chemiluminescence detection system (ECL™ Select western blotting detection reagent: Sigma-Aldrich, MO, United States). Specific protein bands were visualized using the DCP-T300 brother scanner and evaluated using ImageJ software (National Institutes of Health, Bethesda, MD). The full images of electrophoretic blots were represented in Supplementary Materials (Supplementary Material Figure S3).

### Measurement of Neurite Outgrowth and Neurite-Bearing Cells

Neuro-2a cells were performed in neurite outgrowth stimulation assay according to Eik et al. (2012) (Supplementary Materials).

### Knockdown of Teneurin-4 Expression

For Teneurin-4 (Ten-4) knockdown, On-Target Plus small interfering RNA (siRNA), which contains the targeting sequence of Ten-4 (Thermo Fisher Scientific), was used. Specific siRNAs of the Ten-4 gene were designed according to Suzuki et al. (2014), Zhang et al. (2020) (sense: 5′- GAU​UGU​GGC​AAA​CUA​GUA​U-3′, antisense: 5′- AUA​CUA​GUU​UGC​CAC​AAU​C-3′). Transfection of Neuro-2a cells was accomplished using Lipofectamine^®^ 2000 (Invitrogen; Thermo Fisher Scientific, Inc.). The negative control group was transfected with AccuTarget™ Negative Control siRNA (Thermo Fisher Scientific) in Neuro-2a cells. Knockdown efficiency was assessed by quantitative RT-PCR (Supplementary Material Figure S1, Supplementary Materials).

### Statistical Analysis

All experiments were performed at least in triplicate. The data are shown as the mean ± SEM and were analyzed with GraphPad Prism 6. A comparison between the control and treatments was analyzed by one-way ANOVA following Bonferroni’s method (post hoc). Differences were considered significant at the *p* ≤ 0.001 level.

## Results and Discussion

### Phytochemical Constituents of Anacardium Occidentale L. Extracts

**FIGURE 1:**
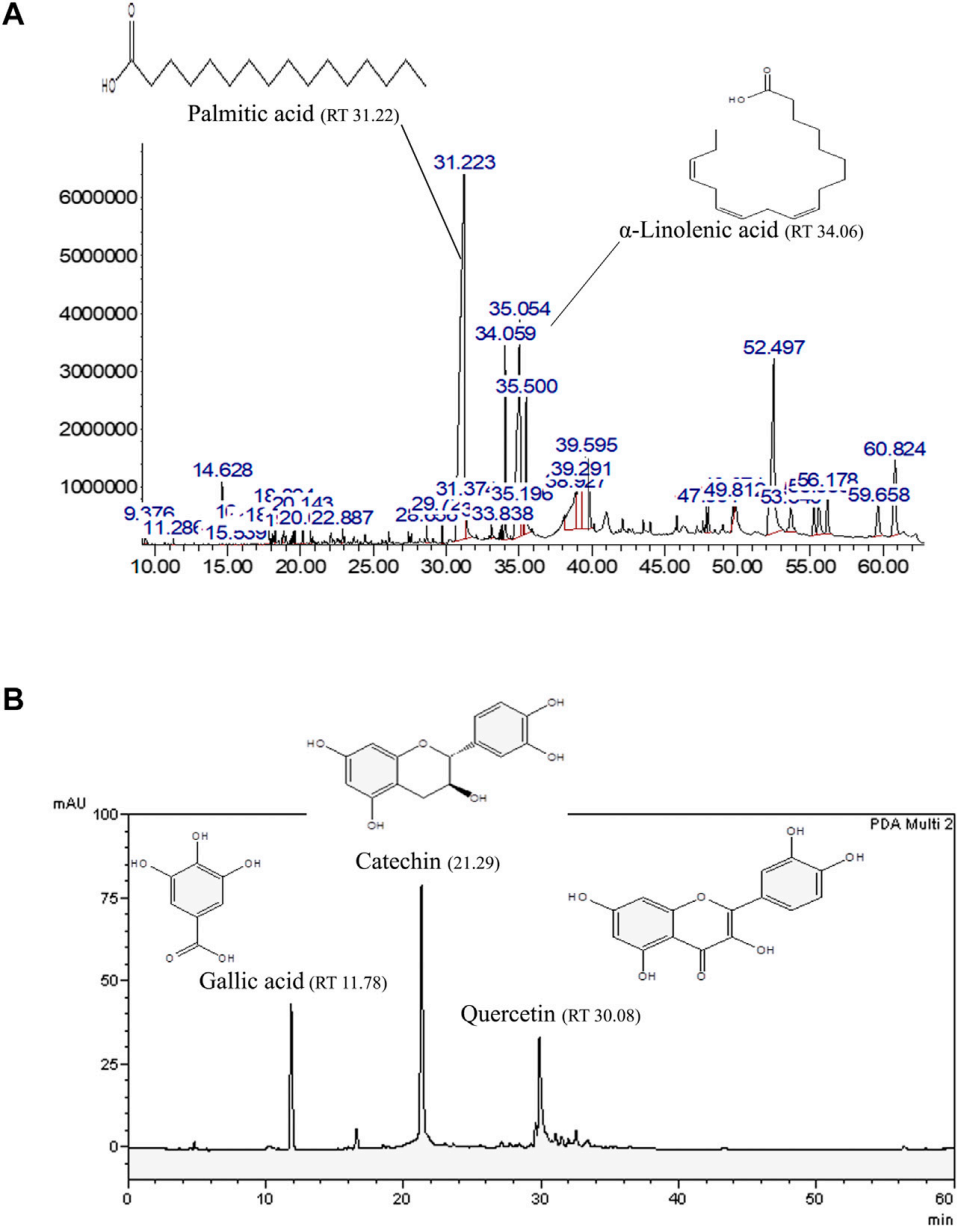
Representative bioactive compounds in AO extracts. **(A)** GLC-MS profile of the AO hexane extract and **(B)** HPLC chromatograms of the AO methanol extract with the chemical structures of their main constituents.

In our previous study, AO hexane and methanol extracts demonstrated oxidative stress resistance properties in a *C. elegans* model (Duangjan et al., 2019a). Thus, hexane and methanol extracts were explored in the present study. GLC-MS profiles represented the main compounds in the AO hexane extract, which were identified as palmitic acid (8495.95 mg/100 g of crude extract) and α-linolenic acid (4073.13 mg/100 g of crude extract) (Figure 1A). Moreover, HPLC showed the presence of bioactive compounds in AO methanol extract that were identified as gallic acid (305.92 mg/100 g of crude extract), catechin (1924.13 mg/100 g of crude extract) and quercetin (707.10 mg/100 g of crude extract) (Figure 1B). These results were consistent with our previous study (Duangjan et al., 2019a).

**GRAPHICAL ABSTRACT:**
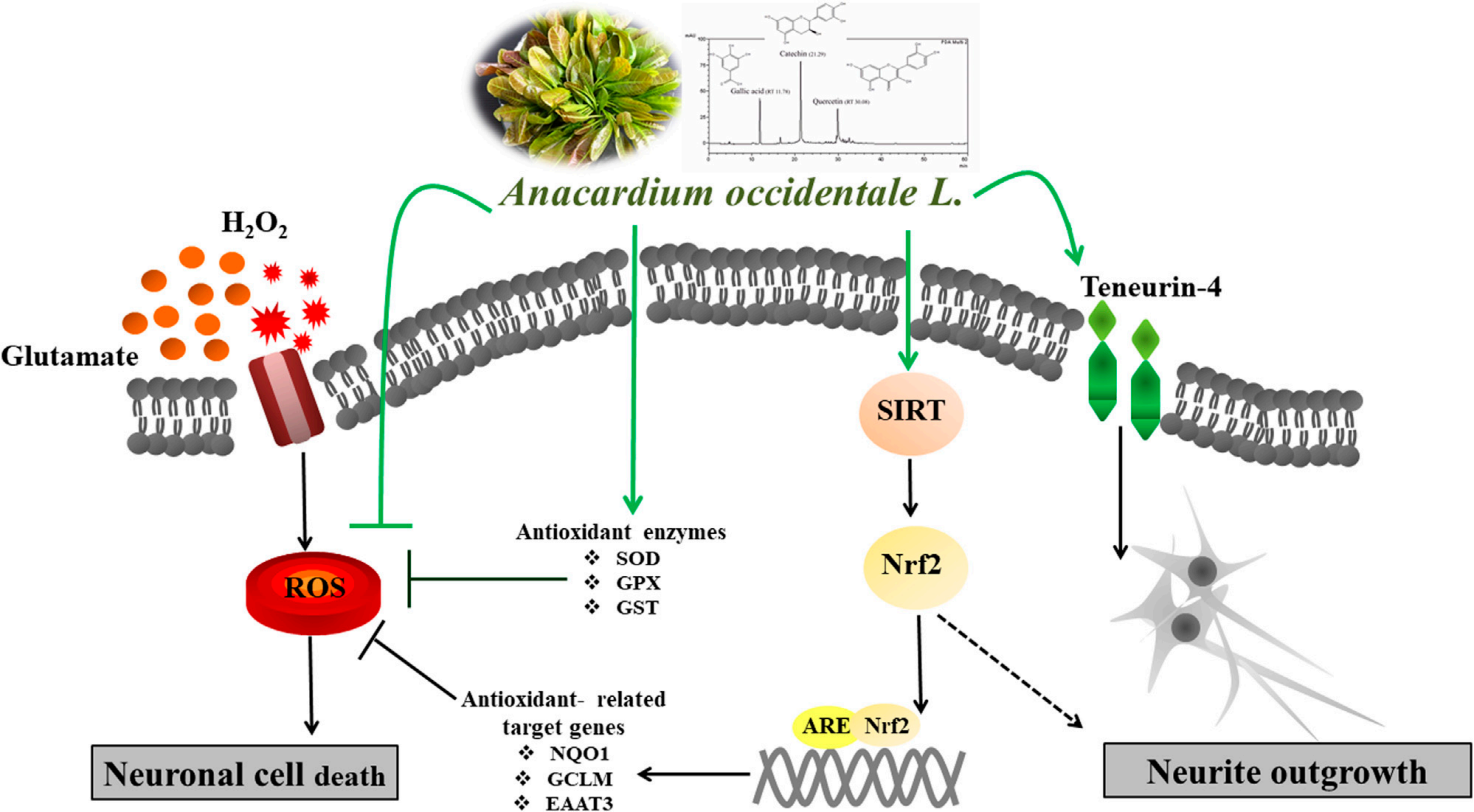


### Cytotoxicity of Anacardium Occidentale L. Extracts on HT22 and Neuro-2a Cells

**FIGURE 2:**
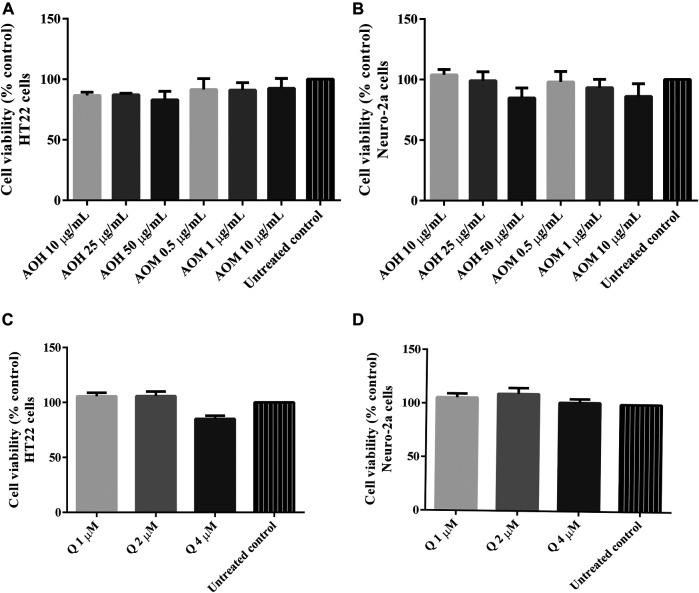
Cytotoxicity effect of AO extracts in neuronal (HT22 and Neuro-2a) cells. The effects of AO extracts on cell viability of HT22 **(A)** and Neuro-2a **(B)** cells. The effects of quercetin on cell viability of HT22 **(C)** and Neuro-2a **(D)** cells. Cells were treated with different concentrations of the extracts or compounds for 48 h following MTT assay. The results are expressed as the means ± SEM of independent experiments (*n* = 3); *p* ≤ 0.001 compared to the untreated control.

Cytotoxic activity of the extracts were tested in HT22 and Neuro-2a cells to show that the extracts were innocuous to a normal cells line. We found that the treatment with different concentrations of AO extracts for 48 h did not change the cell viability of HT22 and Neuro-2a cells compared to the untreated control (Figures 2A,B). Results indicated that the AO extracts were relatively non-cytotoxic at the tested doses in HT22 and Neuro-2a cells.

### Effect of Anacardium Occidentale L. Extracts on H_2_O_2_/Glutamate-Induced Cytotoxicity in HT22 and Neuro-2a Cells

**FIGURE 3:**
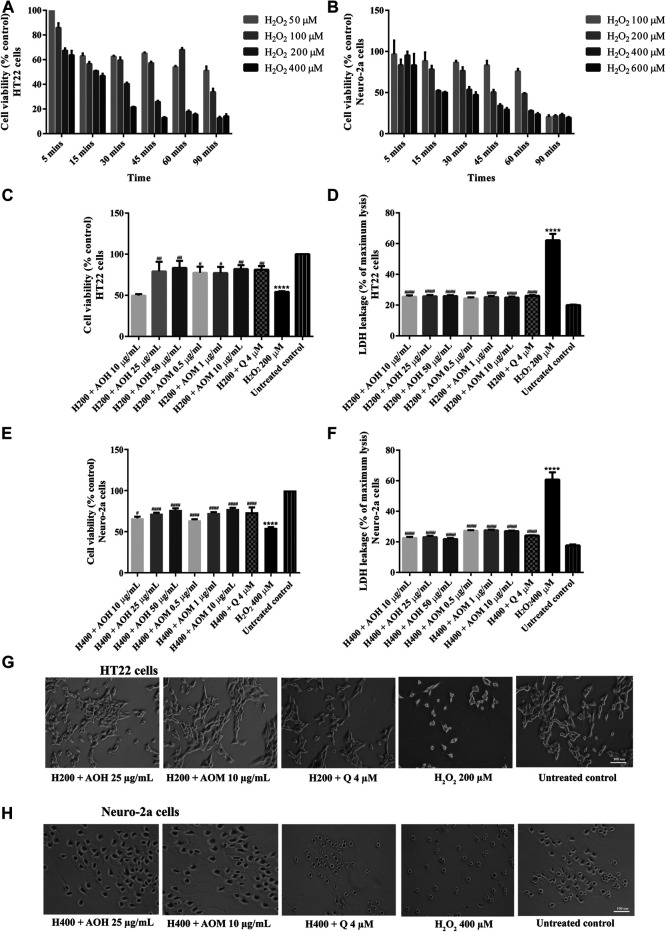
Protective effects of AO extracts on H_2_O_2_-induced toxicity in HT22 and Neuro-2a cells. Cells were exposed to various concentrations of H_2_O_2_ for different times in HT22 **(A)** and Neuro-2a cells, cell viability was measured by MTT assay **(B)**. Cell viability of HT22 **(C,D)** and Neuro-2a cells **(E,F)**, cell morphology of HT22 **(G)** and Neuro-2a **(H)** after treatment with different concentrations of AO extracts. Cell morphology was observed under microscope at 5× magnification. Samples were treated with AO extracts for 48 h and exposed to H_2_O_2_ (H200: 200 µM H_2_O_2_, H400: 400 µM H_2_O_2_) for 15 min to induce toxicity. The results are expressed as the means ± SEM of independent experiments (*n* = 3). ****p < 0.0001 compared to the untreated control; ^#^ *p* < 0.05, ^##^ *p* < 0.01, ^###^ *p* < 0.001 and ^####^ *p* < 0.0001, compared to the group exposed to H_2_O_2_ only.

To investigate the protective effects on AO extracts against the H_2_O_2_/glutamate -induced cytotoxicity in HT22 and Neuro-2a cells, cell viability were measured. To select the appropriate concentration of H_2_O_2_, the cells were exposed to various concentrations of H_2_O_2_ for 5–90 min. Cell viability of HT22 and Neuro-2a exposure to H_2_O_2_ (200 µM for 15 min, HT22 cells; 400 µM for 15 min, Neuro-2a cells) decreased by 50% when compared to the untreated control (Figures 3A,B). However, the viability of both cells pretreated with AO extracts had significantly lower H_2_O_2_-induced cell death compared to that of the cells exposed to H_2_O_2_ alone (Figures 3C,E).

**FIGURE 4:**
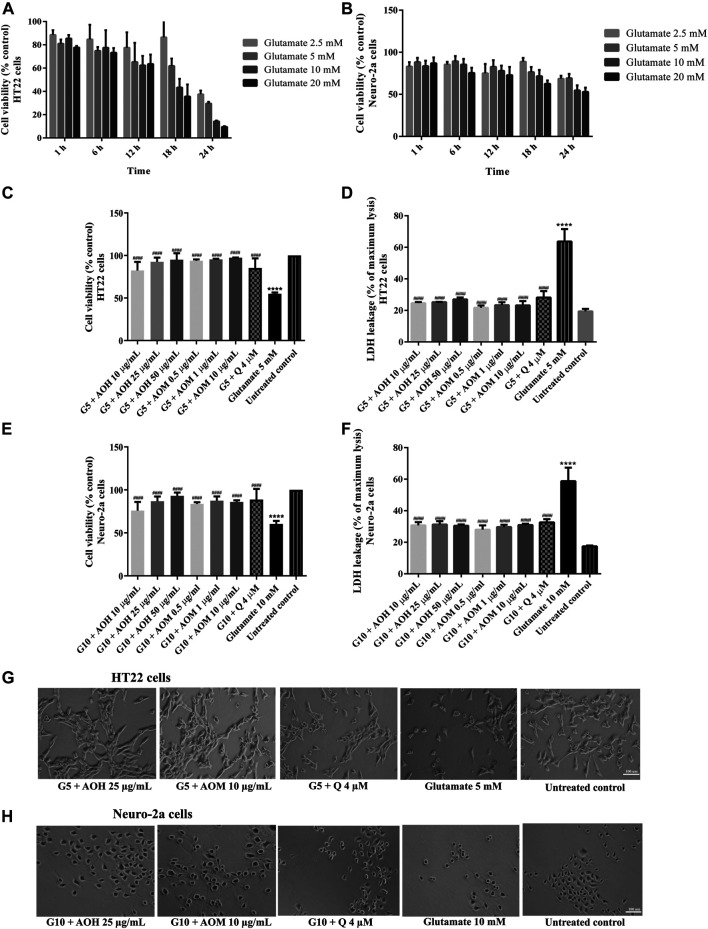
Protective effects of AO extracts on glutamate-induced toxicity in HT22 and Neuro-2a cells. Cells were exposed to various concentrations of glutamate for different times in HT22 **(A)** and Neuro-2a cells, cell viability was measured by MTT assay **(B)**. Cell viability of HT22 **(C,D)** and Neuro-2a cells **(E,F)**, cell morphology of HT22 **(G)** and Neuro-2a **(H)** after treatment with different concentrations of AO extracts. Cell morphology was observed under microscope at 5× magnification. Samples were treated with AO extracts for 48 h and exposed to glutamate (G5: 5 mM glutamate, G10: 10 mM glutamate) for 18 h (HT22 cells) or 24 h (Neuro-2a cells) to induce toxicity. The results are expressed as the means ± SEM of independent experiments (*n* = 3). ****p < 0.0001 compared to the untreated control; ^#^ *p* < 0.05, ^##^ *p* < 0.01, ^###^ *p* < 0.001 and ^####^ *p* < 0.0001, compared to the group exposed to glutamate only.

In a complementary set of experiments, the optimal condition for glutamate-induced toxicity was elucidated. The cells were exposed to various concentrations of glutamate for 1–24 h. Cell viability of HT22 and Neuro-2a exposure to glutamate (5 mM for 18 h, HT22 cells; 10 mM for 24 h, Neuro-2a cells) decreased by 50% when compared to the untreated control (Figures 4A,B). Surprisingly, the viability of the both cells pretreated with AO extracts had significantly lower glutamate-induced cell death compared to that of the cells exposed to glutamate alone (Figures 4C,E).

Results were in a similar range as the quercetin positive control which is a well-known neuroprotective compound (Park et al., 2019), and were confirmed by LDH assay (Figures 3D,F, 4D,F) as well as morphological examination (Figures 3G,H, 4G,H). Results suggest that AO extracts exert a potent neuroprotective effect against cytotoxicity induced by H_2_O_2_/glutamate in neuronal cells.

### Effect of Anacardium Occidentale L. Extracts on Glutamate-Induced Oxidative Stress in HT22 and Neuro-2a Cells

**FIGURE 5:**
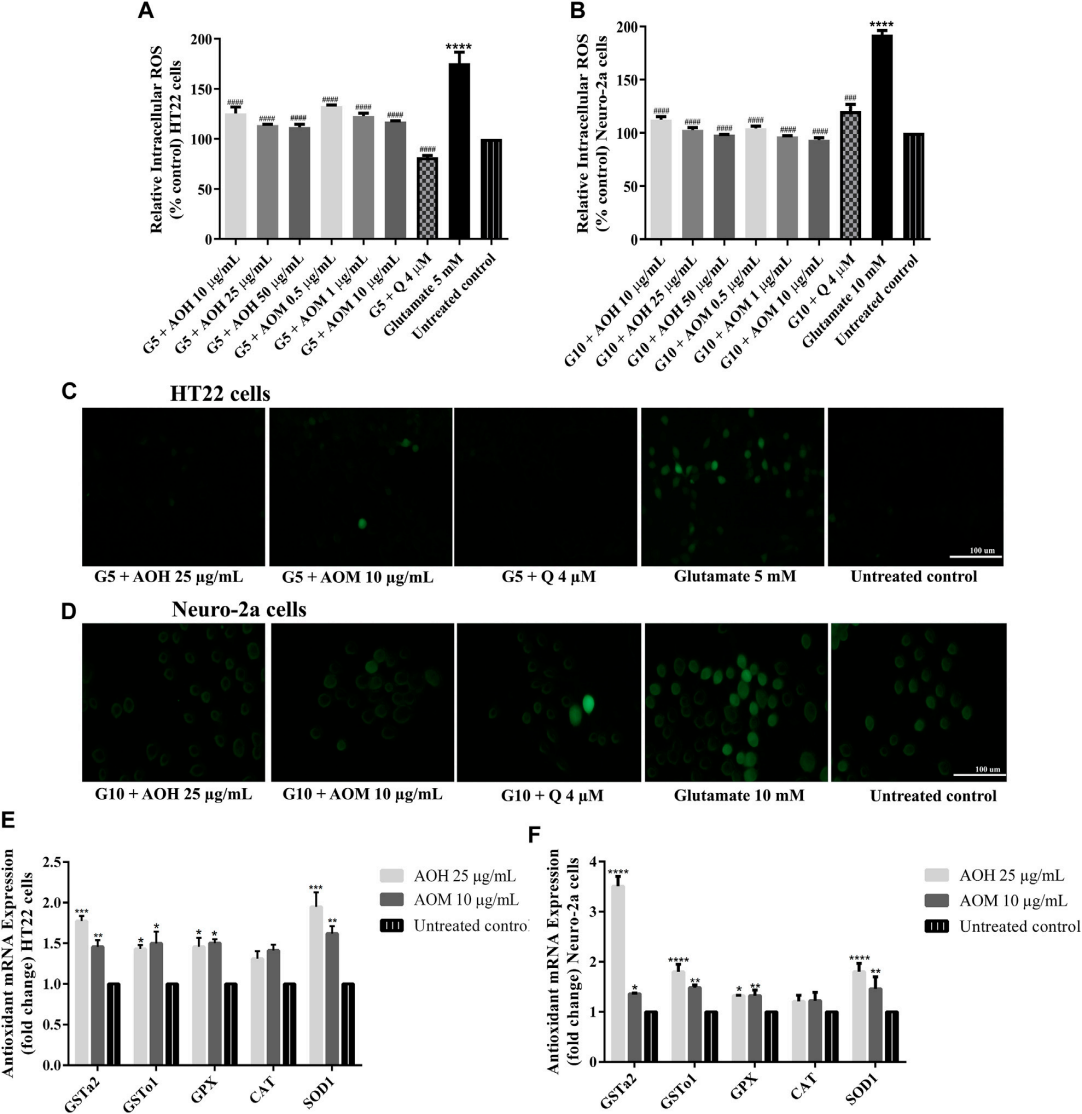
Protective effect of AO extracts on glutamate-induced oxidative stress. AO extract treatment reduced ROS levels in HT22 **(A)** and Neuro-2a **(B)** cells when compared to glutamate-treated cells. Representative fluorescence micrographs of HT22 **(C)** and Neuro-2a **(D)** cells stained with DCFH-DA were observed under a fluorescence microscope (10×) (Representative microscopy images from DCFH-DA, phase contrast and nuclear staining can be found in the Supplementary Material Figure S5. Samples were treated with AO extracts for 48 h and exposed to glutamate (G5: 5 mM glutamate, G10: 10 mM glutamate) for 12 h (HT22 cells) or 18 h (Neuro-2a cells) to induce oxidative stress. AO extract treatment increased endogenous antioxidant gene expression in HT22 **(E)** and Neuro-2a **(F)** cells when compared to untreated control. β-actin was used as the internal control for RT-PCR assay. The results are expressed as the means ± SEM of independent experiments (*n* = 3). *p < 0.05, **p < 0.01, ***p < 0.001 and ****p < 0.0001, compared to the untreated control; #*p* < 0.05, ##*p* < 0.01, ###*p* < 0.001 and ####*p* < 0.0001, compared to the group exposed to glutamate only.

In our previous study, AO extracts exhibited powerful antioxidant activity *in vitro* and *in vivo* (Duangjan et al., 2019a). To investigate whether AO extracts could suppress glutamate-induced oxidative stress, the antioxidant properties of AO extracts in neuronal (HT22 and Neuro-2a) cells were explored. Intracellular ROS level was significantly elevated in HT22 (approximately 1.7 fold) and Neuro-2a (approximately 1.9 fold) cells after exposure to glutamate, compared to the untreated control. Therefore, glutamate-induced cytotoxicity in neuronal (HT22 and Neuro-2a) cells was indeed associated with intracellular ROS increase. However, HT22 and Neuro-2a cells pretreated with AO extracts significantly reduced the elevated levels of ROS in the same range as the quercetin positive control (Figures 5A–D, Supplementary Material Figure S5, Supplementary Materials). Results suggest that AO extracts protect against glutamate-induced cytotoxicity by suppressing intracellular ROS production.

### Effect of Anacardium Occidentale L. Extracts on Gene Expression of Antioxidant Enzymes in HT22 and Neuro-2a Cells

It is well known that glutamate-induced oxidative stress result in neuronal cell death (Fukui et al., 2009). To investigate the protective effects of AO extracts on glutamate-induced oxidative stress through endogenous antioxidant enzymes, we investigated the expression of antioxidant and phase II enzymes, including superoxide dismutase (SOD), catalase (CAT), glutathione peroxidase (GPx), and glutathione-S-transferase (GST) which, have a major role in preventing ROS-mediated cellular damage (Sukprasansap et al., 2017).

Previous results showed that 25 μg/ml AO hexane extract and 10 μg/ml AO methanol extract exhibited a powerful neuroprotective effect in HT22 and Neuro-2a cells (Figures 3–5). Thus, these concentrations were used for the following experiments. HT22 and Neuro-2a cells, pretreated with 25 μg/ml AO hexane extract and 10 μg/ml AO methanol extract, showed significantly increased expression of endogenous antioxidant enzymes including SOD1, GPx, GSTo1, and GSTa2 (Figures 5E,F). These results agree with our previous study that AO extracts also stimulated the expression of stress-response genes including, SOD-3 and GST-4 in *C. elegans* (Duangjan et al., 2019a). However, the expression of the CAT gene was not significantly changed in either HT22 or Neuro-2a (Figures 5E,F). Results demonstrated that the protective effect of AO extracts against glutamate/H_2_O_2_-induced cytotoxicity was achieved not only by suppressing intracellular ROS production, but also through enhancing the expression of endogenous antioxidant enzymes. The neuroprotective effects of AO extracts may occur because of antioxidant activity from bioactive compounds such as palmitic acid (Ghareghani et al., 2017), α-linolenic acid (Lee et al., 2018), gallic acid (Oiram Filho et al., 2018), catechin (Herges et al., 2011; Zhang et al., 2020), and quercetin (Yang et al., 2014; Khan et al., 2018; Park et al., 2019), which were found in several studies to have neuroprotective properties.

### Effect of Anacardium Occidentale L. Extracts on Cellular Antioxidant Defense Via SIRT1/Nrf2-Dependent Response

**FIGURE 6:**
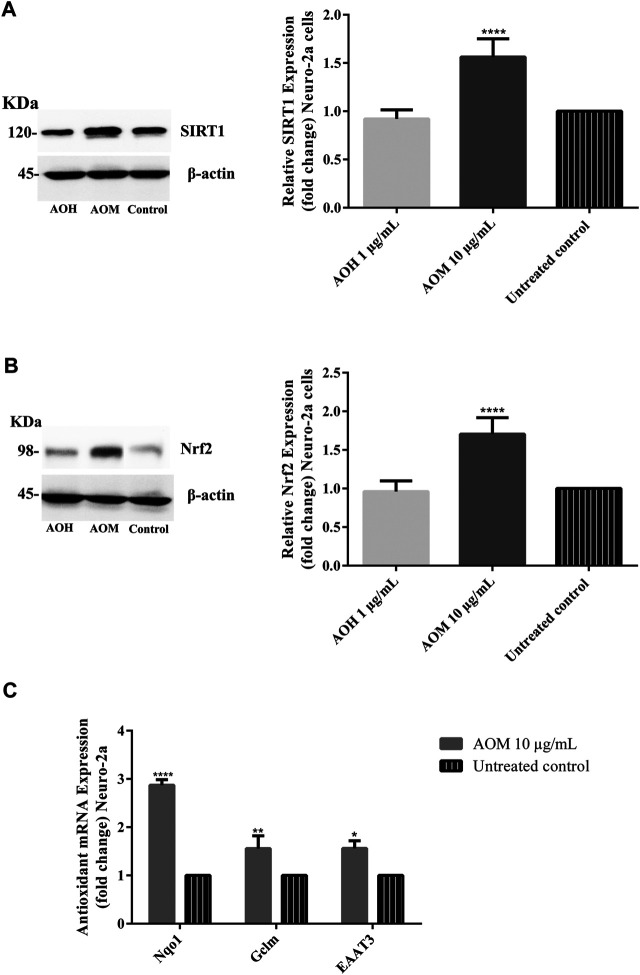
Effect of AO extracts on SIRT1/Nrf2 signaling pathway. AO methanol extract treatment increased the SIRT1 **(A)**, Nrf2 expression **(B)** and antioxidant-related target genes **(C)** in Neuro-2a cells when compared to the untreated control. Samples were treated with AO extracts for 48 h. Whole-cell lysates were subjected to western blot analysis of the SIRT1 and Nrf2 levels after AO extract treatment. β-actin was used as an endogenous loading control for western blot assay and internal control for RT-PCR assay. All data were normalized to endogenous control levels and the results are expressed as the means ± SEM of independent experiments (*n* = 3). *p < 0.05, **p < 0.01, ***p < 0.001 and ****p < 0.0001, compared to the untreated control.

To investigate the underlying neuroprotective mechanism of AO extracts, we studied the SIRT1/Nrf2 signaling pathway. Pretreatment with 10 μg/ml AO methanol extract significantly increased the expressions of SIRT1 and Nrf2 (mRNA and protein levels) (Figures 6A,B). However, 1 μg/ml AO hexane extract did not cause a significant change in SIRT1 and Nrf2 expression compared to the untreated control (Figures 6A,B). To further extend our study, the effects of AO methanol extract on antioxidant-related target genes that are regulated by the SIRT1/Nrf2 signaling pathway were elucidated. In addition, 10 μg/ml AO methanol extract also induced antioxidant-related target genes including NQO1, GCLM, and EAAT3 in Neuro-2a cells (Figure 6C). Collectively, the findings demonstrate that AO methanol extract promotes antioxidant defense in Neuro-2a cells may be involved in the SIRT1/Nrf2 signaling pathway.

Recent studies demonstrated that phenolic antioxidants and aromatic compounds can activate ARE and induce the Nrf2/ARE signaling pathway (Jung and Kwak, 2010; Khan et al., 2018; Duangjan et al., 2019b; Yang et al., 2020), AO methanol extract contains phenolic (flavonoid glycoside) compounds including gallic acid, catechin and quercetin. Thus protective effects mediated by SIRT1/Nrf2 signaling pathway may be due to phenolic compounds in AO methanol extract. Moreover, a previous study also reported the oxidative stress resistance properties of AO methanol extract via the SKN-1/Nrf-2 signaling pathways in *C. elegans* (Duangjan et al., 2019a).

### Effect of Anacardium Occidentale L. Extracts on Neurite Outgrowth Activity in Neuro-2a Cells

Neuritogenesis or neurite outgrowth is a process in the differentiation of neurons that plays a central role in neuronal development and the formation of synapses (Lam et al., 2016). The induction of neuronal differentiation is one of the neuroprotective factors. The effects of AO extracts on neurite outgrowth activity were explored in this study. Optimal concentrations of AO extracts (1 μg/ml AO hexane and 10 μg/ml AO methanol) were used for neurite outgrowth activity (Supplementary Material Figure S2, Supplementary Materials).

**FIGURE 7:**
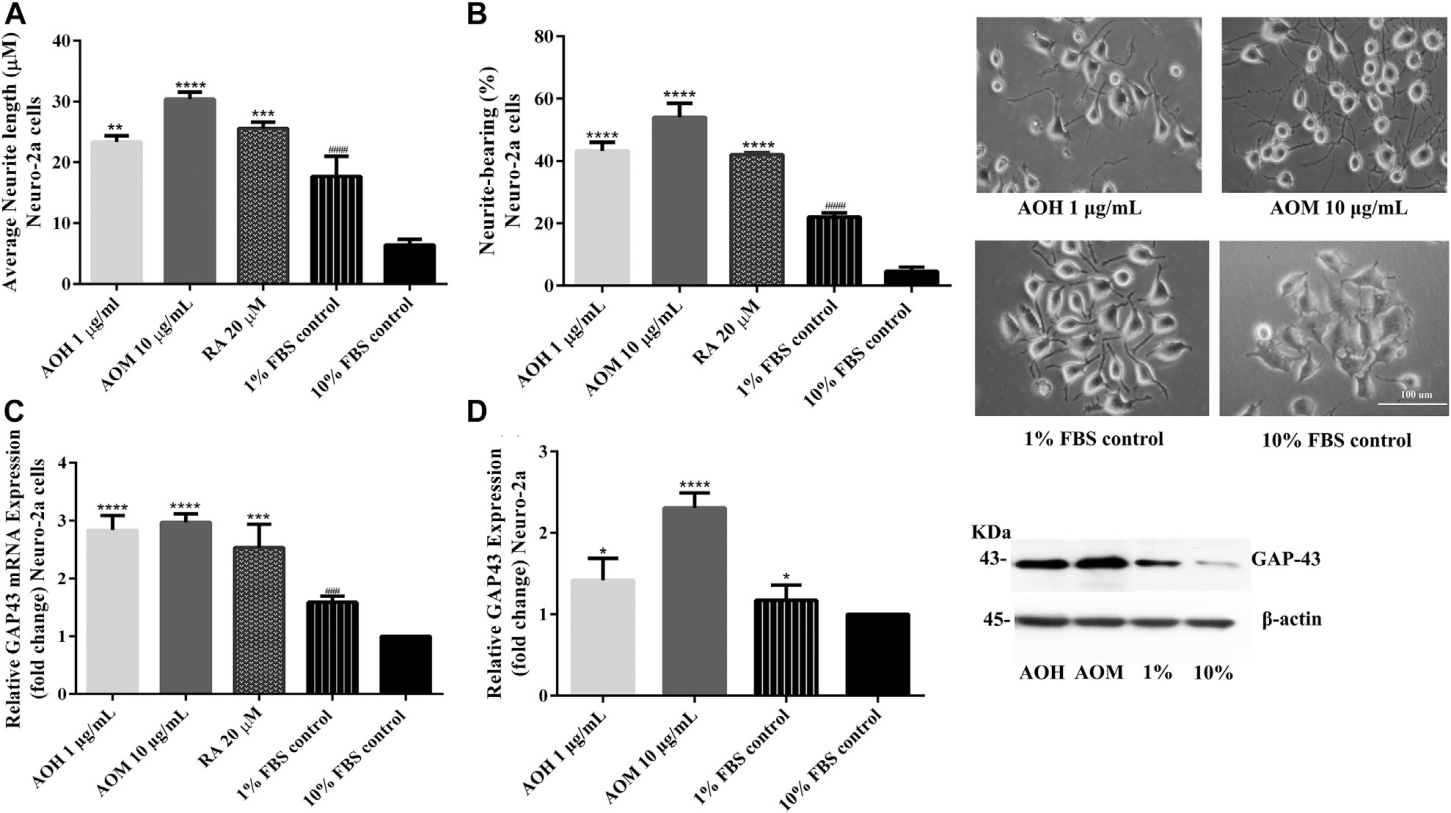
Effect of AO extracts on neurite outgrowth. AO extract treatment increased the average of neurite lengths **(A)** and the percentage of neurite-bearing cells **(B)** in Neuro-2a cells. Cell morphology of Neuro-2a cells was observed under a microscope at 10× magnification. Relative expression levels of mRNA **(C)** and protein **(D)** GAP-43 in Neuro-2a cells. Samples were treated with AO extracts for 48 h. Whole-cell lysates were subjected to western blot analysis of the GAP43 level after AO extract treatment. β-actin was used as an endogenous loading control for western blot assay and internal control for RT-PCR assay. All data were normalized to 10% FBS control level and the results are expressed as the means ± SEM of independent experiments (*n* = 3). *p < 0.05, **p < 0.01, ***p < 0.001 and ****p < 0.0001 compared to the 1% FBS control; ^###^ *p* < 0.001 and ^####^ *p* < 0.0001 compared to the 10% FBS control.

To investigate the effect of AO extracts on neurite outgrowth activity in Neuro-2a cells, the cells were maintained in a low-serum medium (DMEM supplemented with 1% FBS). Neuro-2a cells, that were treated with 1 μg/ml AO hexane extract, exhibited significantly increased neurite lengths (23.36 µm) and neurite bearing cells (43.25%) when compared to the 1% FBS control (neurite length, 17.68 µm; neurite bearing cells, 22.06%) (Figures 7A,B). In addition, Neuro-2a cells that were treated with 10 μg/ml AO methanol extract, showed significantly increased neurite length (30.38 µm) and neurite bearing cells (54.06%) when compared to the 1% FBS control (Figures 7A,B). The neurite outgrowth inducing effects were similar to those of retinoic acid, which is a well-known inducer of neuronal differentiation (Kumar and Katyal, 2018).

To further confirm neurite outgrowth activities, GAP-43 expression, a marker of neurite outgrowth, was measured. Neuro-2a cells treated with AO extracts had significantly increased GAP-43 expression (mRNA and protein levels) when compared to 1% FBS control (Figures 7C,D). Results suggest that AO extracts have an effect on neuritogenesis in Neuro-2a cells. These results agree with several recent studies regarding the neurodegeneration properties of α-linolenic acid, gallic acid (Siddiqui et al., 2019), catechin (Herges et al., 2011) and quercetin (Chan et al., 2018; Katebi et al., 2019).

### Involvement of Signaling Pathway in Anacardium Occidentale L. Extracts Induced Neurite Outgrowth in Neuro-2a Cells

**FIGURE 8:**
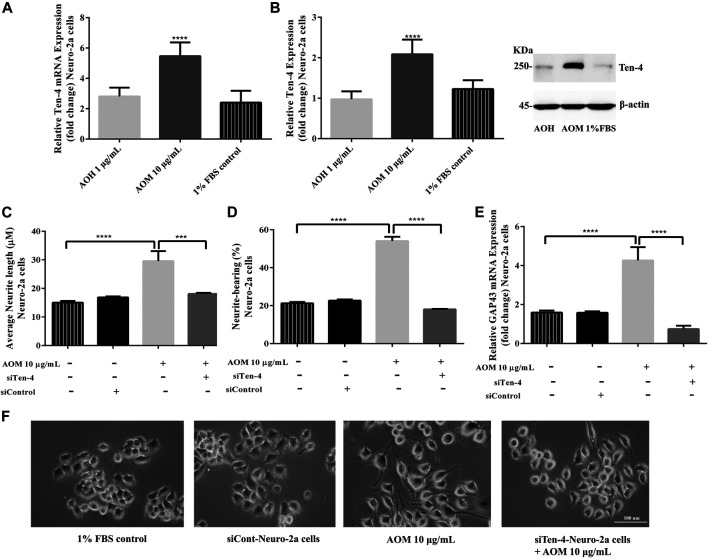
Effect of AO extracts on Ten-4-mediated neurite outgrowth. AO methanol extract treatment increased expression level of Ten-4 mRNA **(A)** and protein **(B)**. AO methanol extract failed to induced neurite length **(C)** and neurite-bearing cells **(D)** in siTen-4-Neuro-2a cells. Results were confirmed by GAP43 mRNA expression **(E)**. Cell morphology of Neuro-2a cells was observed under a microscope at 10× magnification **(F)**. Samples were treated with AO extracts for 48 h. Whole-cell lysates were subjected to western blot analysis at the Ten-4 level after AO extract treatment. β-actin was used as endogenous loading control for western blot assay and internal control for RT-PCR assay. All data were normalized to 10% FBS control levels in siCont-Neuro-2a cells and the results are expressed as the means ± SEM of independent experiments (*n* = 3). *p < 0.05, **p < 0.01, ***p < 0.001 and ****p < 0.0001 compared to the 1% FBS control.

To investigate whether Ten-4 expression is involved in AO extracts-induced neurite growth in Neuro-2a cells, mRNA and protein expression levels of Ten-4 were examined. Neuro-2a cells treated with 10 μg/ml AO methanol extract exhibited significantly increased expression of Ten-4 mRNA and corresponding protein levels (Figures 8A,B). However, 1 μg/ml AO hexane extract was inactive (Figures 8A,B).

To confirm the role of Ten-4 in AO methanol extract-induced neurite outgrowth in Neuro-2a cells, Ten-4 siRNA (siTen-4) were used. When Ten-4 expression was knocked down by siTen-4, AO methanol extract failed to induce neurite length and neurite bearing cells in Neuro-2a cells agreeing with GAP-43 expression (Figures 8C–F). Taken together, the findings demonstrate that AO methanol extracts promote neurite outgrowth in Neuro-2a cells mediated by the Teneurin-4 transmembrane protein.

The available evidences suggest that SIRT1 and Nrf2 are also involved in neuroprotection and neurogenesis (Sugino et al., 2010; Liang et al., 2019). Thus, the neuroprotective and neurogenesis effects of AO methanol extract may be mediated by the SIRT1/Nrf2 signaling pathway. Nevertheless, further studies are needed to investigate the effects of AO methanol extract on neurite outgrowth via the SIRT1/Nrf2 signaling pathway.

AO contains a number of total phenolic compounds such as flavonoids, anthocyanins, and tannins which are therapeutically recognized in the treatment of several age-related diseases (Salehi et al., 2019). Moreover, AO contains phenolic lipids and anacardic acids, which have been reported in antimicrobial, antitumoral and antioxidant activities (Salehi et al., 2019; Siracusa et al., 2020).

The high concentrations of anacardic acids exhibited toxicity effects in bacteria and melanoma cells by inhibiting bacterial cell growth (Muroi and Kubo, 1996) and cancer cell proliferation (Radde et al., 2016; Tan et al., 2017). In contrast, the proper concentrations appeared antioxidant activities to modulate the immune responses and angiogenesis (Gomes Júnior et al., 2020).

Our previous study observed that AO extracts can effectively protect *C. elegans* against severe oxidative stress and attenuate intracellular ROS levels at moderate concentrations (Duangjan et al., 2019a). Higher concentrations of AO extracts cannot attenuate ROS levels in the worms, possibly due to a pro-oxidant activity of the plant extracts according to a high level of anacardic acids concentration (Duangjan et al., 2019a).

In this study, we observed that AO extracts can effectively protect neuronal cells against severe oxidative stress and attenuate intracellular ROS levels. We suggested that the optimal concentration of AO extract is need for antioxidant and protective properties (Supplementary Material Figure S4). The neuroprotective effects of AO extracts are consistent with antioxidant properties against neurotoxicity of anacardic acids (Salehi et al., 2019), gallic acid (Oiram Filho et al., 2018), catechin (Herges et al., 2011; Zhang et al., 2020) and quercetin (Yang et al., 2014; Khan et al., 2018; Park et al., 2019).

Phytotherapy has many potentially significant advantages associated with synergistic interactions such as increased efficiency and reduced undesirable effects (Haroun and Al-Kayali, 2016). The synergistic interactions of bioactive compounds in AO extracts may involve neuroprotective effects and neurite outgrowth properties. There is the imitation for using the crude extracts in this study. It cannot conclude that the therapeutic effects are from a single compound or the synergistic interactions of bioactive compounds. However, there are several strengths for using the crude extracts such as the synergistic effect of the compounds and using the plants as dietary supplements. Further experiments of isolated bioactive components from AO extracts need to be done to confirm our interpretation. Moreover, the investigation focusing on the active components of AO extracts e.g., anacardic acids, gallic acid, catechin and quercetin, are interesting topics to clarify the neuroprotective properties of AO extracts.

## Conclusion

In conclusion, these findings demonstrate the neuroprotective effects and neurite outgrowth properties of AO extracts in cultured neuronal (HT22 and Neuro-2a) cells. AO extracts exhibit neuroprotective effects against glutamate/H_2_O_2_-induced oxidative toxicity in neuronal cells which are mediated via inhibition of ROS accumulation, up-regulation of endogenous antioxidant enzymes, and the increase of the SIRT1/Nrf2 signaling. Significantly, AO extracts promoted neurite outgrowth via the up-regulation of Ten-4 expression. These results suggest that the leaf extracts have an interesting neuritogenesis and neuroprotective potential against glutamate/H_2_O_2_-mediated toxicity and could be a potential therapeutic candidate for neurodegenerative diseases. However, further studies focusing on the active components of AO extracts are crucial to verify the exact mechanisms involved in order to support the therapeutic potential of the plant extracts for alternative or adjunct treatment of neurodegenerative diseases.

## Data Availability

The raw data supporting the conclusions of this article will be made available by the authors, without undue reservation, to any qualified researcher.

## Glossary

AO: anacardium occidentale L.
AOH: AO hexane extract
AOD: AO dichloromethane extract
AOM: AO methanol extract
AD: alzheimer’s disease
SOD: superoxide dismutase
CAT: catalase
GPx: glutathione peroxidase
GST: glutathione-S-transferase
DMSO: dimethyl sulfoxide
GLC-MS: gas/liquid chromatography-mass spectrometry
HPLC: high-performance liquid chromatography
ROS: reactive oxygen species
RNS: reactive nitrogen species
H2DCF-DA: 2,7-dichlorofluorescein diacetate
SIRT: sirtuin 1
Nrf2: nuclear factor-E2-related factor 2
AREs: antioxidant response elements
GCLM: glutamate cysteine ligase complex modifier subunit
NQO1: NAD(P)H, quinone oxidoreductase 1;
EAAT3: excitatory amino acid transporter 3
SOD-3: superoxide dismutase-3
GST-4: glutathione S-transferase 4
Ten-4: teneurin-4
GAP-43: growth associated protein 43
PBS: phosphate buffer saline
DMEM: dulbecco’s modified eagle’s medium
HamF12: ham’s nutrient mixture F12
LDH: lactate dehydrogenase
MTT: 3-(4,5-dimethylthiazol-2-yl)-2,5-diphenyltetrazolium bromide
FBS: fetal bovine serum
H2O2: hydrogen peroxide
PCR: polymerase chain reaction
DNA: deoxyribonucleic acid
RNA: ribonucleic acid
