# Supplementary figures and images for: Anacardium Occidentale L. Leaf Extracts Protect Against Glutamate/H2O2-Induced Oxidative Toxicity and Induce Neurite Outgrowth: The Involvement of SIRT1/Nrf2 Signaling Pathway and Teneurin 4 Transmembrane Protein
Source: Front Pharmacol. 2021 Apr 23;12:627738. doi: 10.3389/fphar.2021.627738 (PMC8114061; doi:10.3389/fphar.2021.627738)

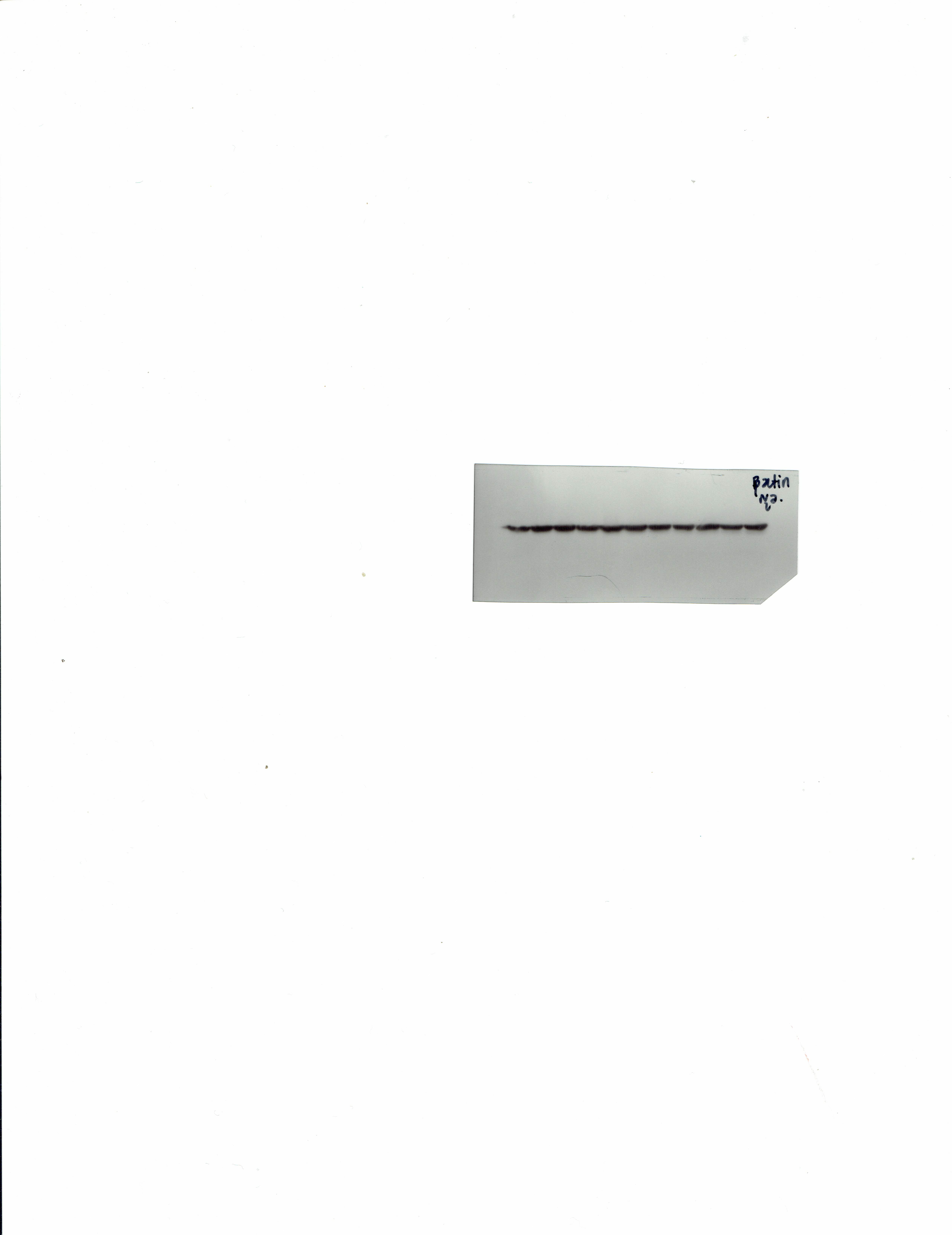

Supplement: Supplementary file 1 [file DataSheet2.ZIP › AO Neuro Manuscript_WB Figures/A1_Bactin.jpg]

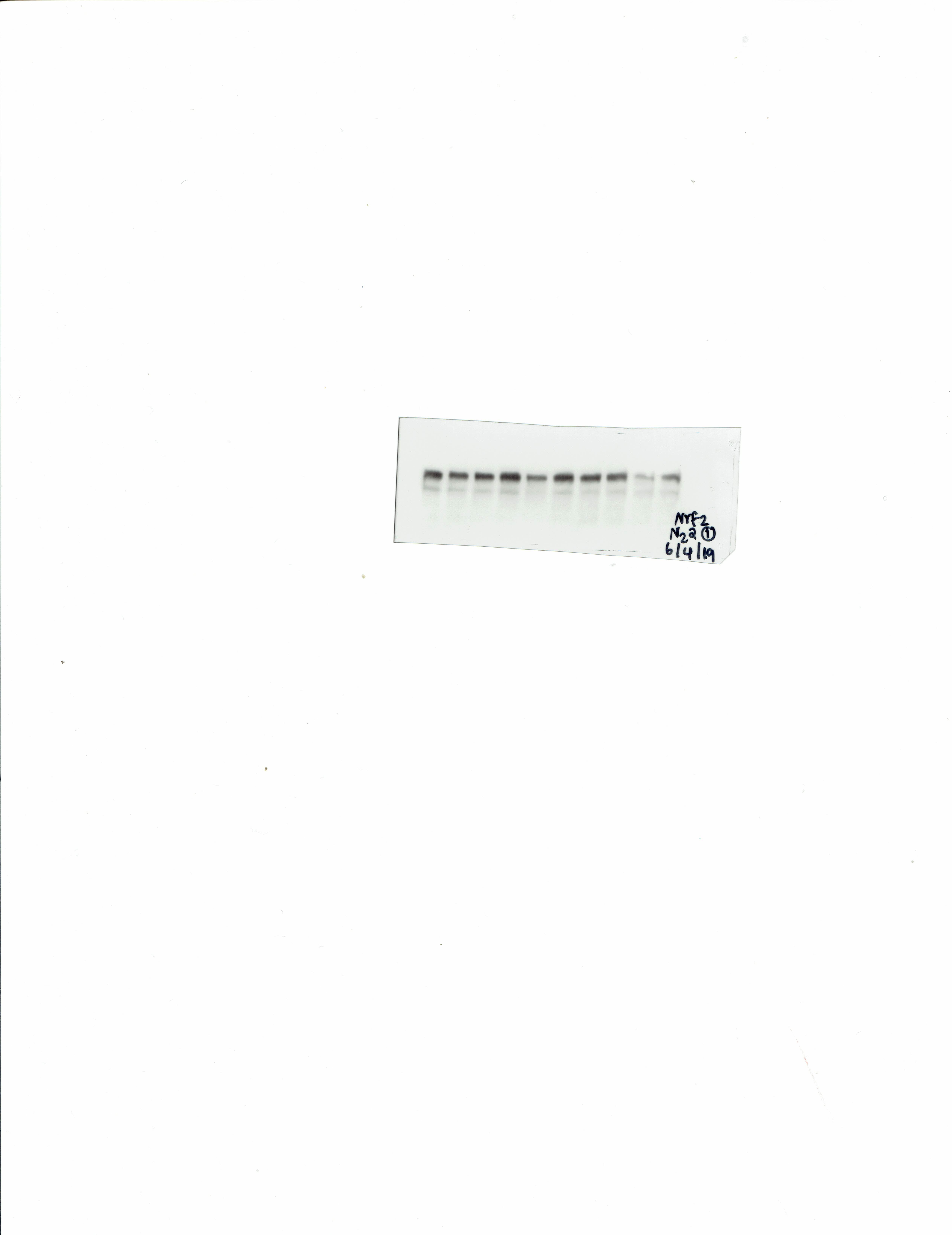

Supplement: Supplementary file 1 [file DataSheet2.ZIP › AO Neuro Manuscript_WB Figures/A1_Nrf2.jpg]

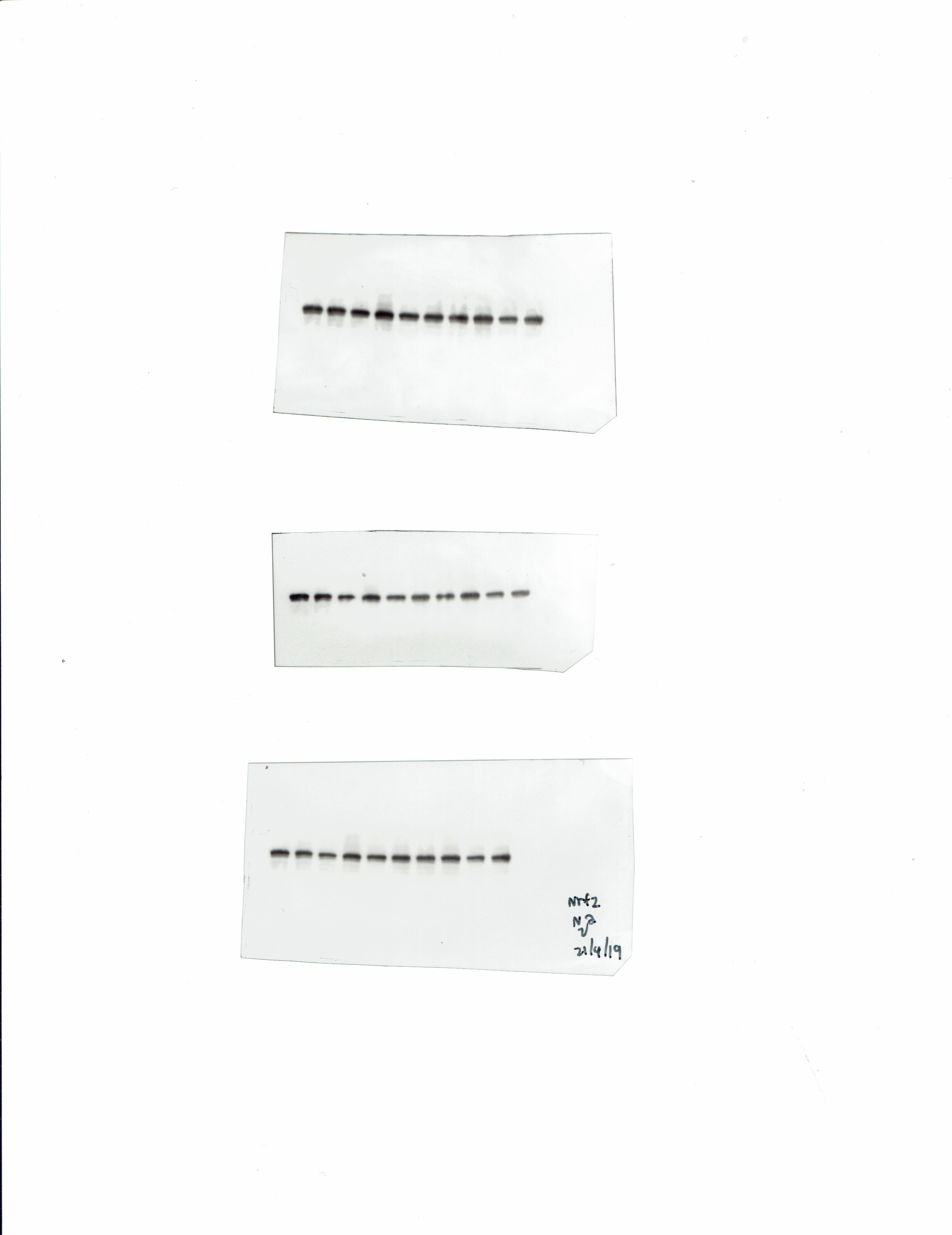

Supplement: Supplementary file 1 [file DataSheet2.ZIP › AO Neuro Manuscript_WB Figures/A2 and A3_ Nrf2.jpg]

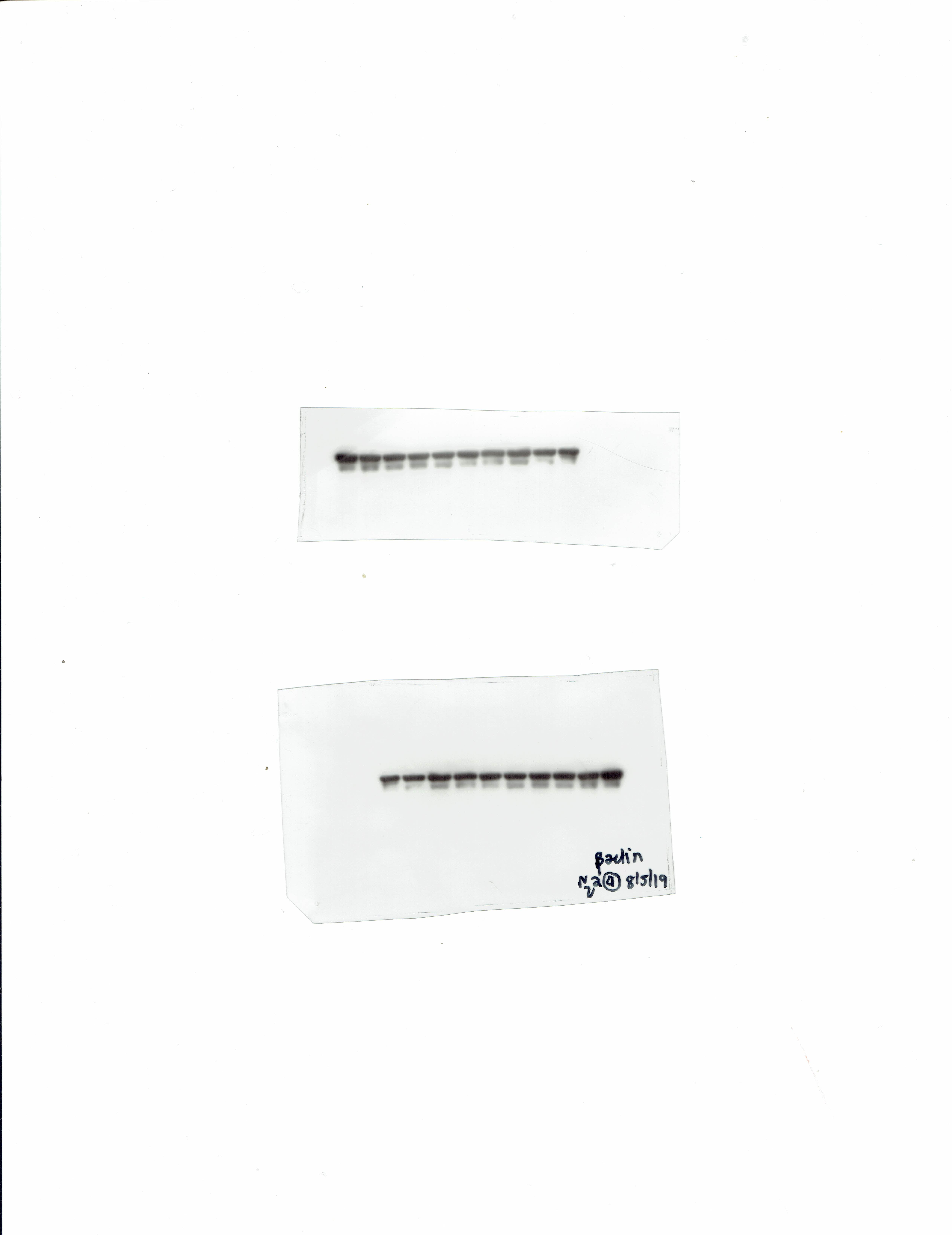

Supplement: Supplementary file 1 [file DataSheet2.ZIP › AO Neuro Manuscript_WB Figures/A3_Bactin.jpg]

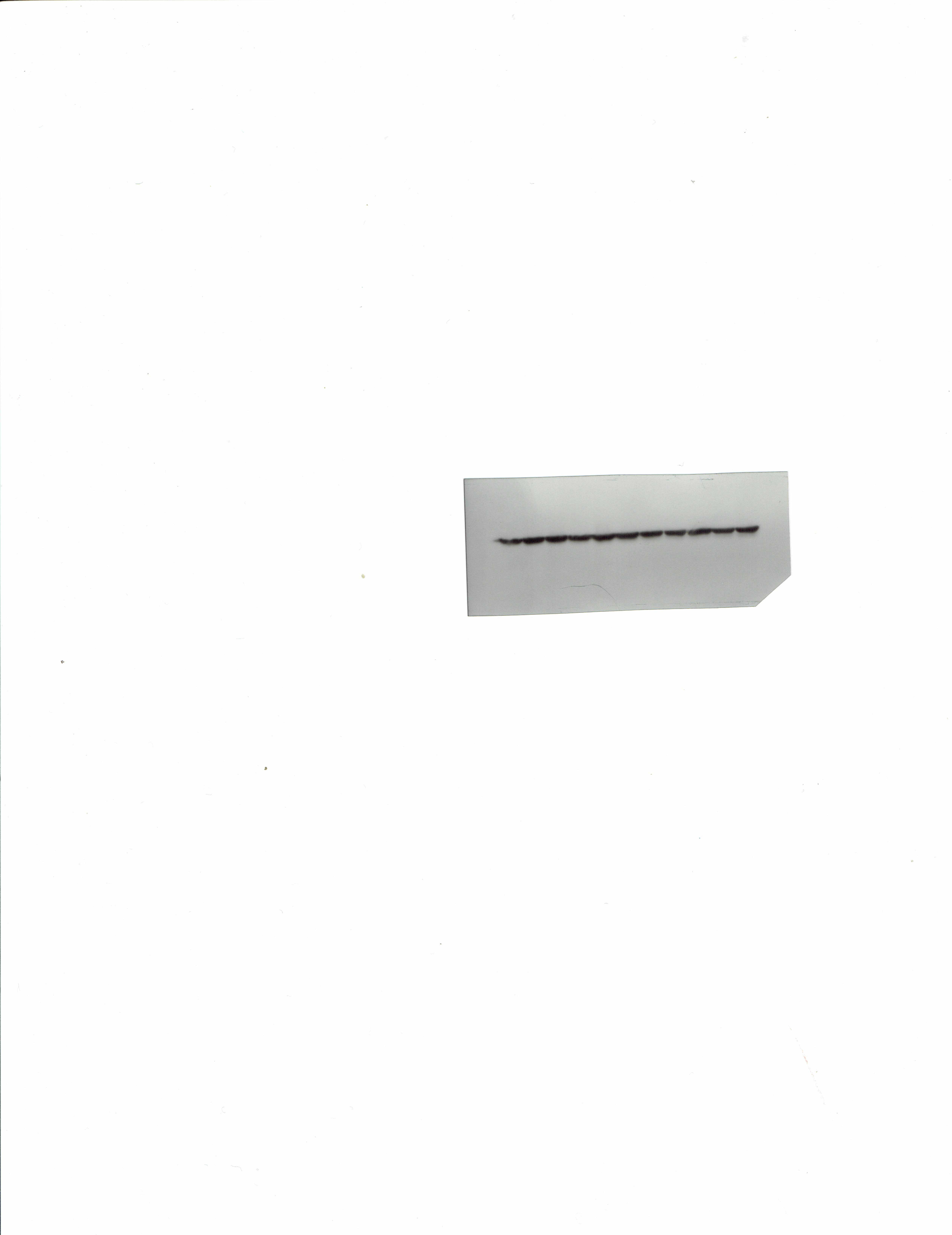

Supplement: Supplementary file 1 [file DataSheet2.ZIP › AO Neuro Manuscript_WB Figures/B1_Bactin.jpg]

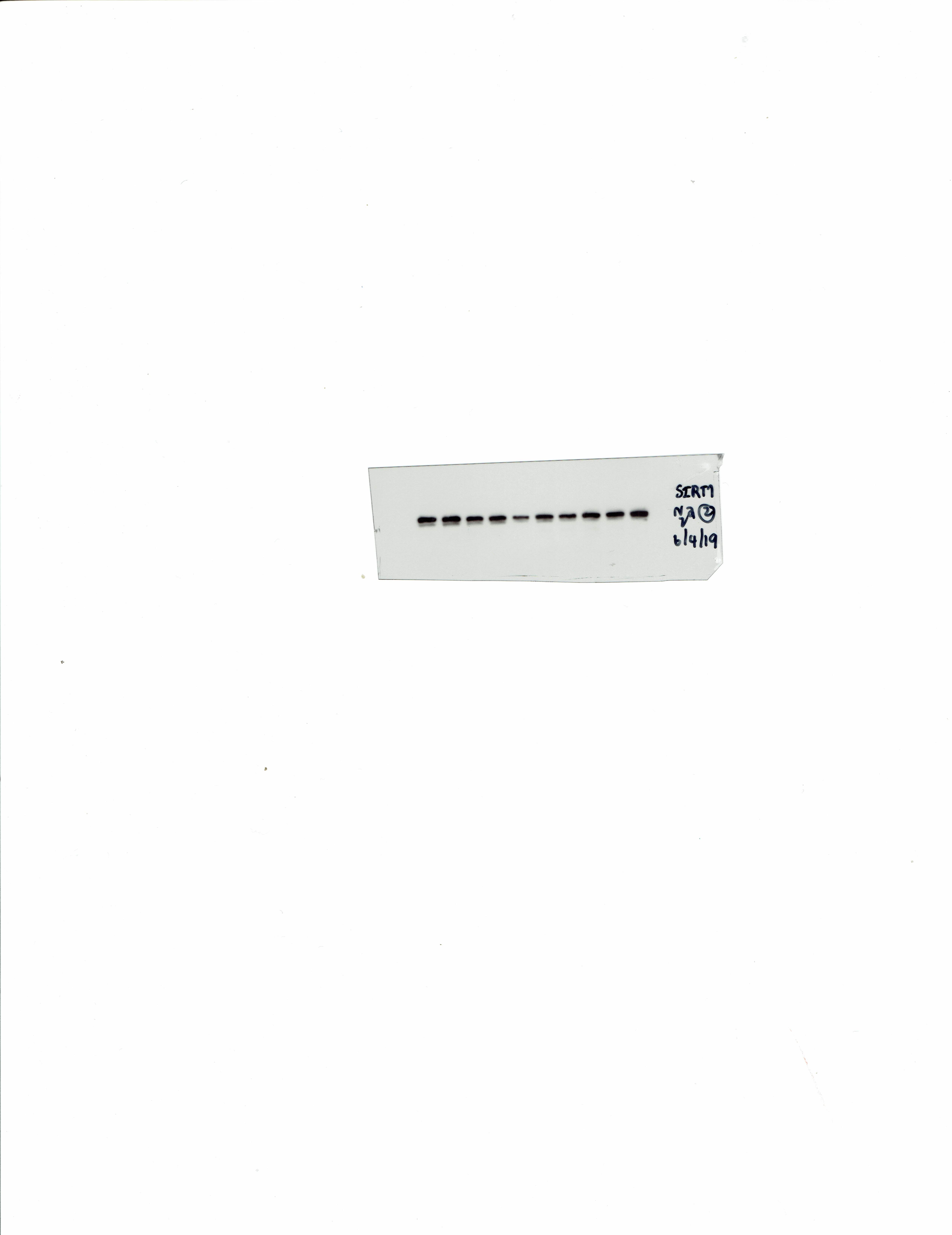

Supplement: Supplementary file 1 [file DataSheet2.ZIP › AO Neuro Manuscript_WB Figures/B1_Sirt1.jpg]

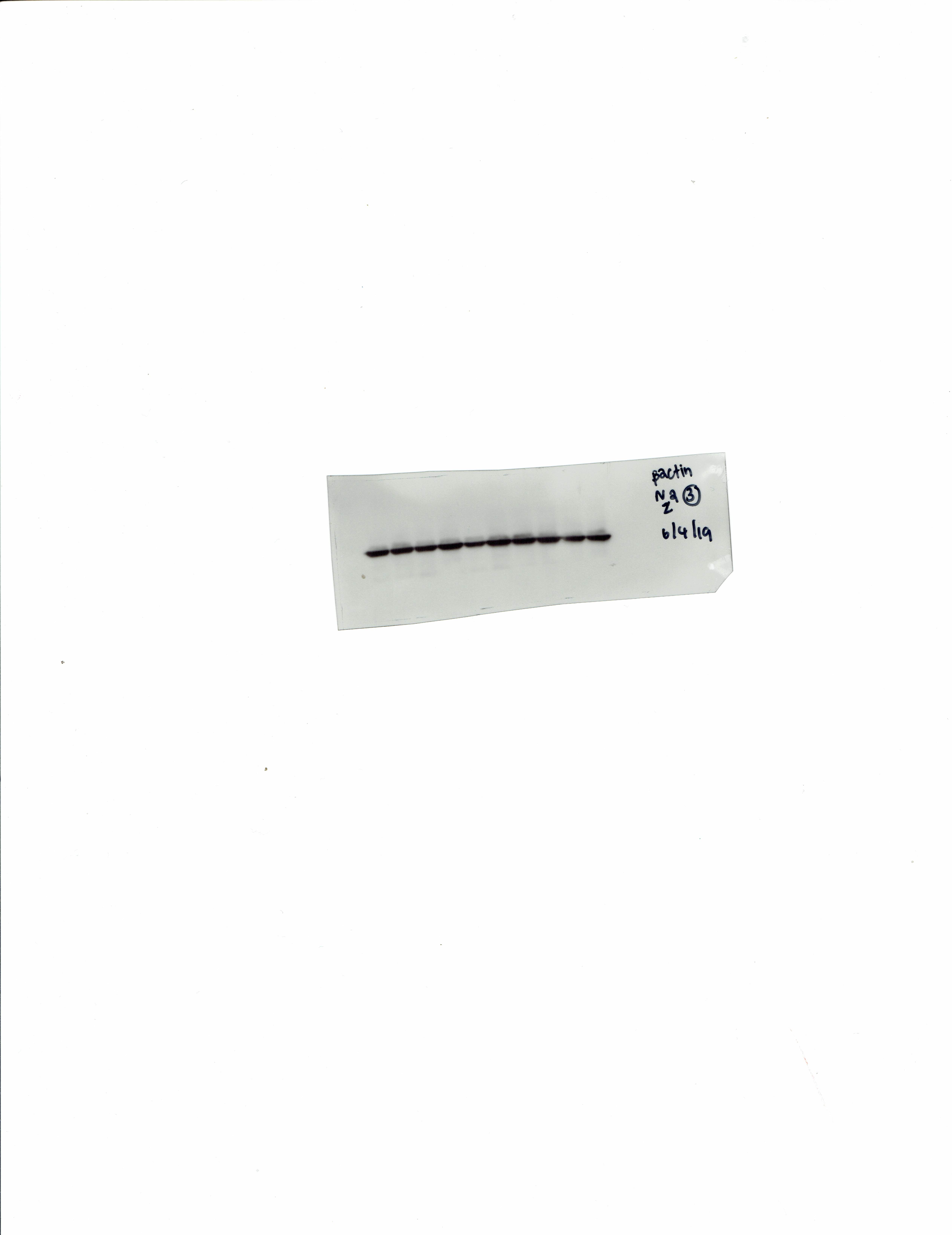

Supplement: Supplementary file 1 [file DataSheet2.ZIP › AO Neuro Manuscript_WB Figures/B2_Bactin.jpg]

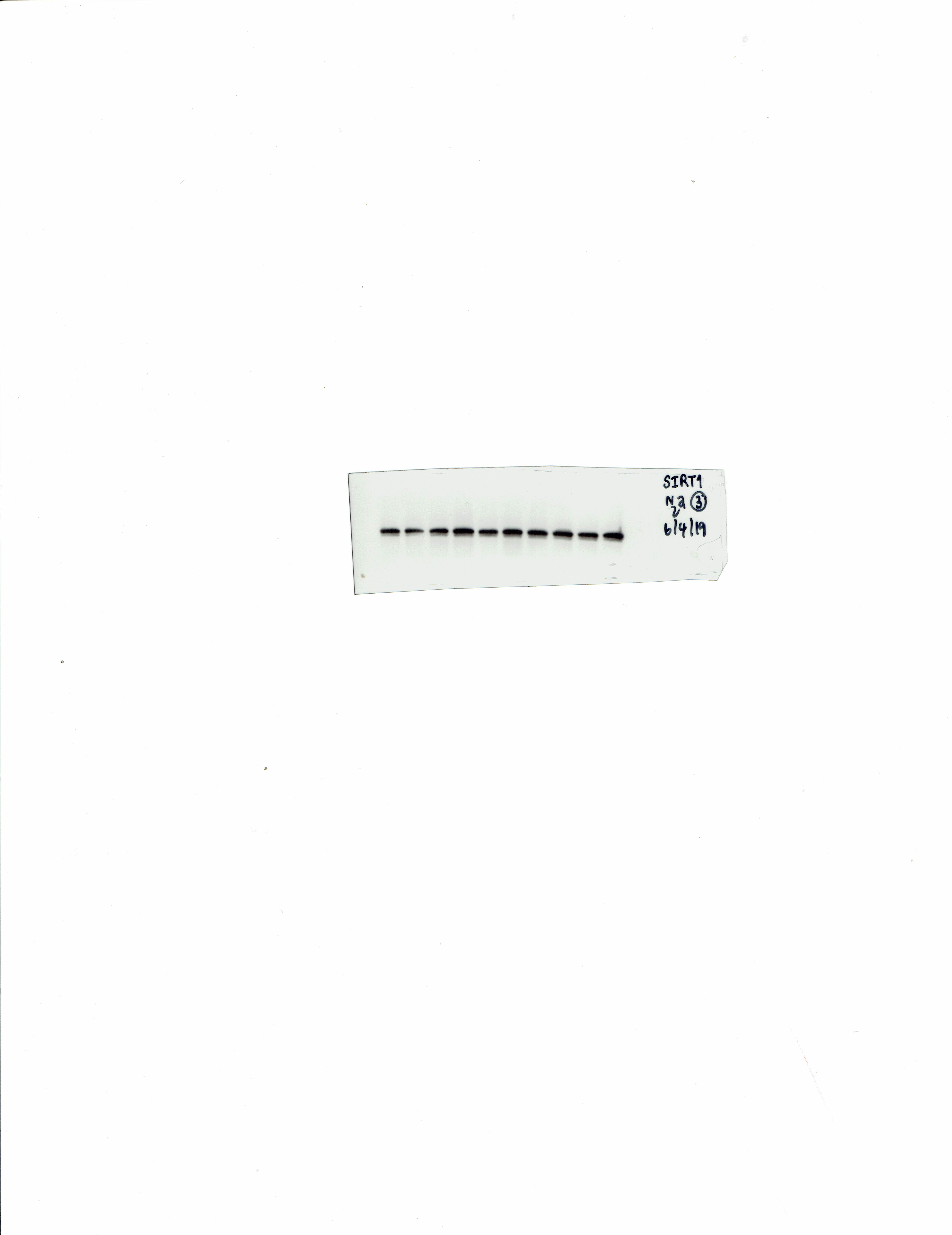

Supplement: Supplementary file 1 [file DataSheet2.ZIP › AO Neuro Manuscript_WB Figures/B2_Sirt1.jpg]

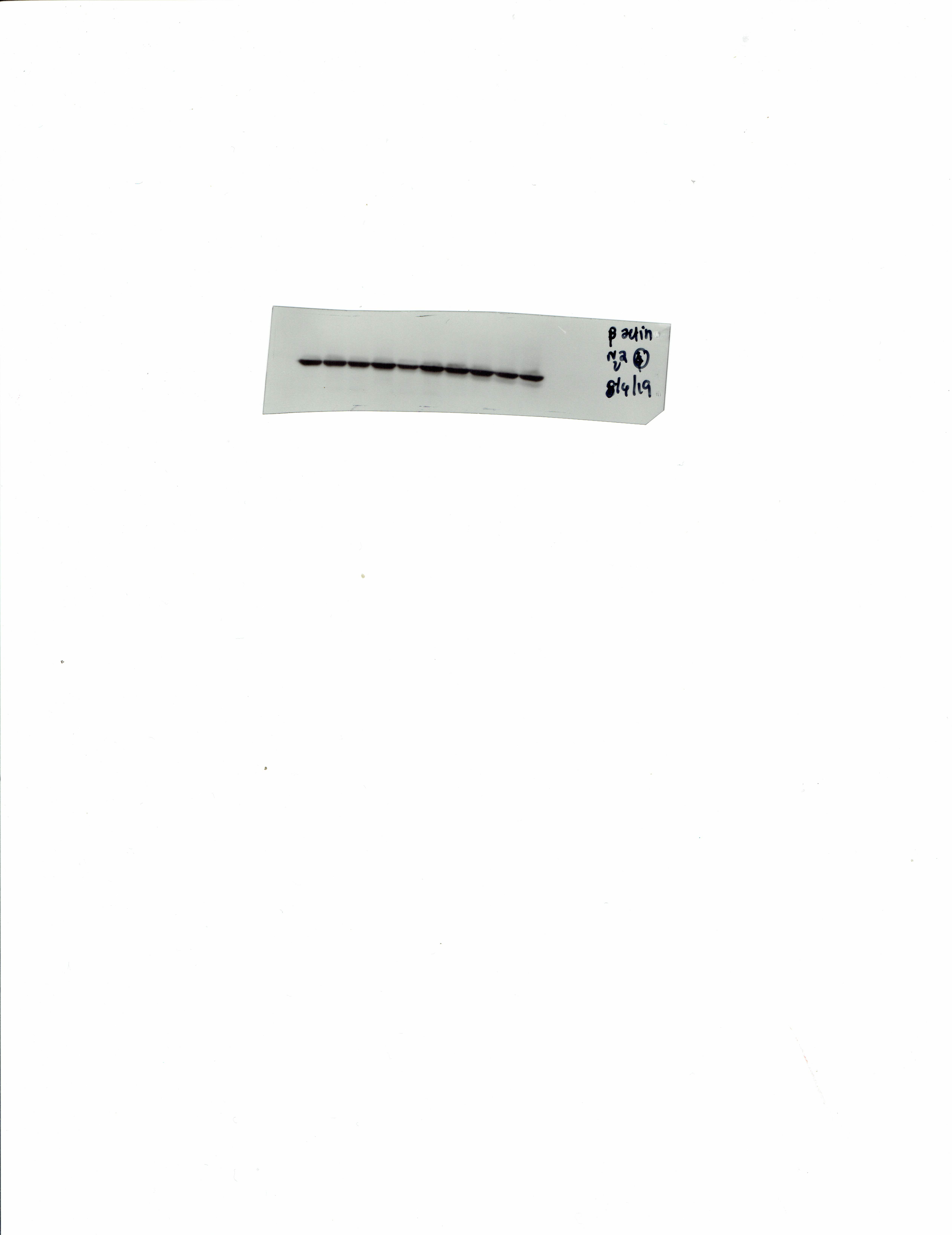

Supplement: Supplementary file 1 [file DataSheet2.ZIP › AO Neuro Manuscript_WB Figures/B3_Bactin.jpg]

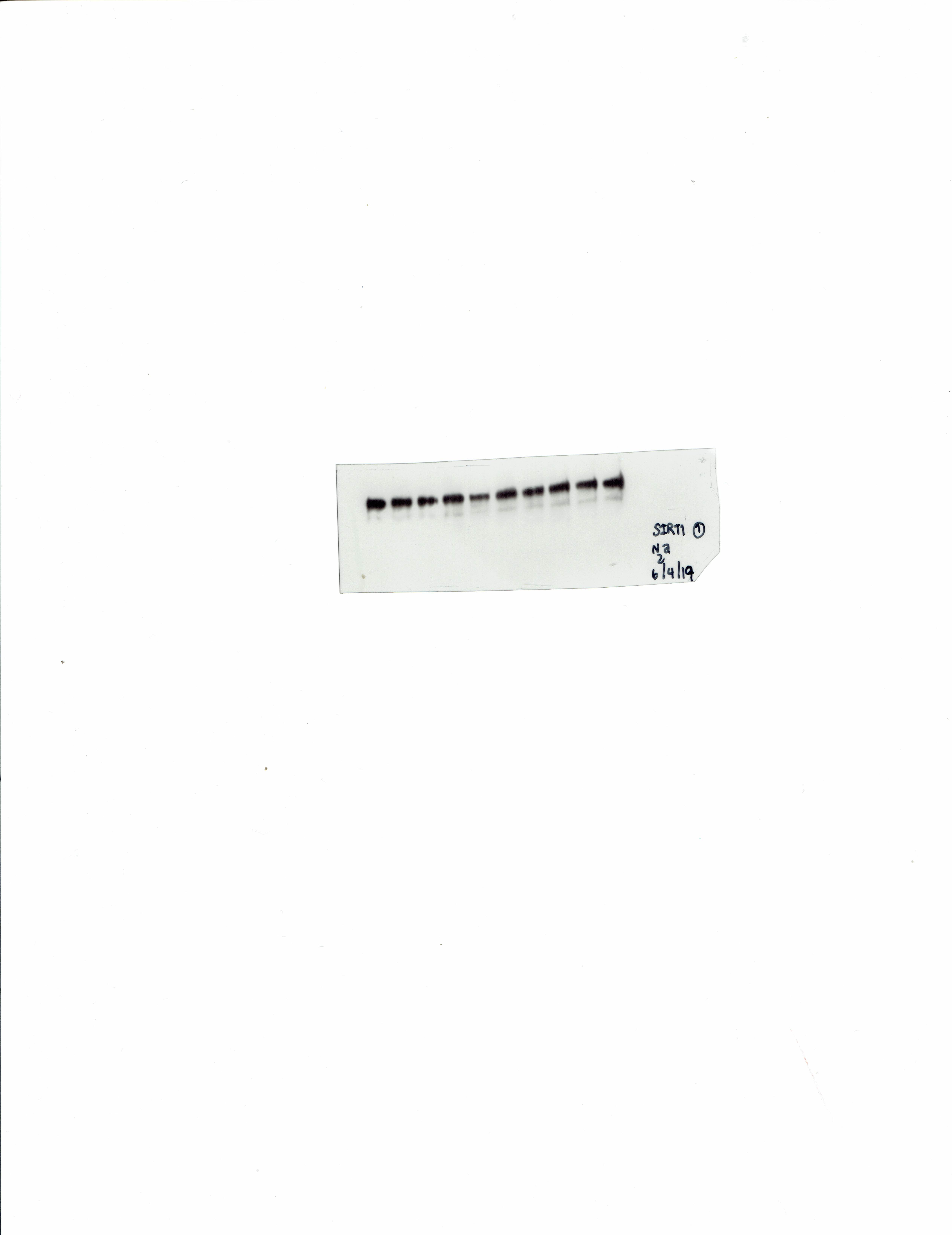

Supplement: Supplementary file 1 [file DataSheet2.ZIP › AO Neuro Manuscript_WB Figures/B3_Sirt1.jpg]

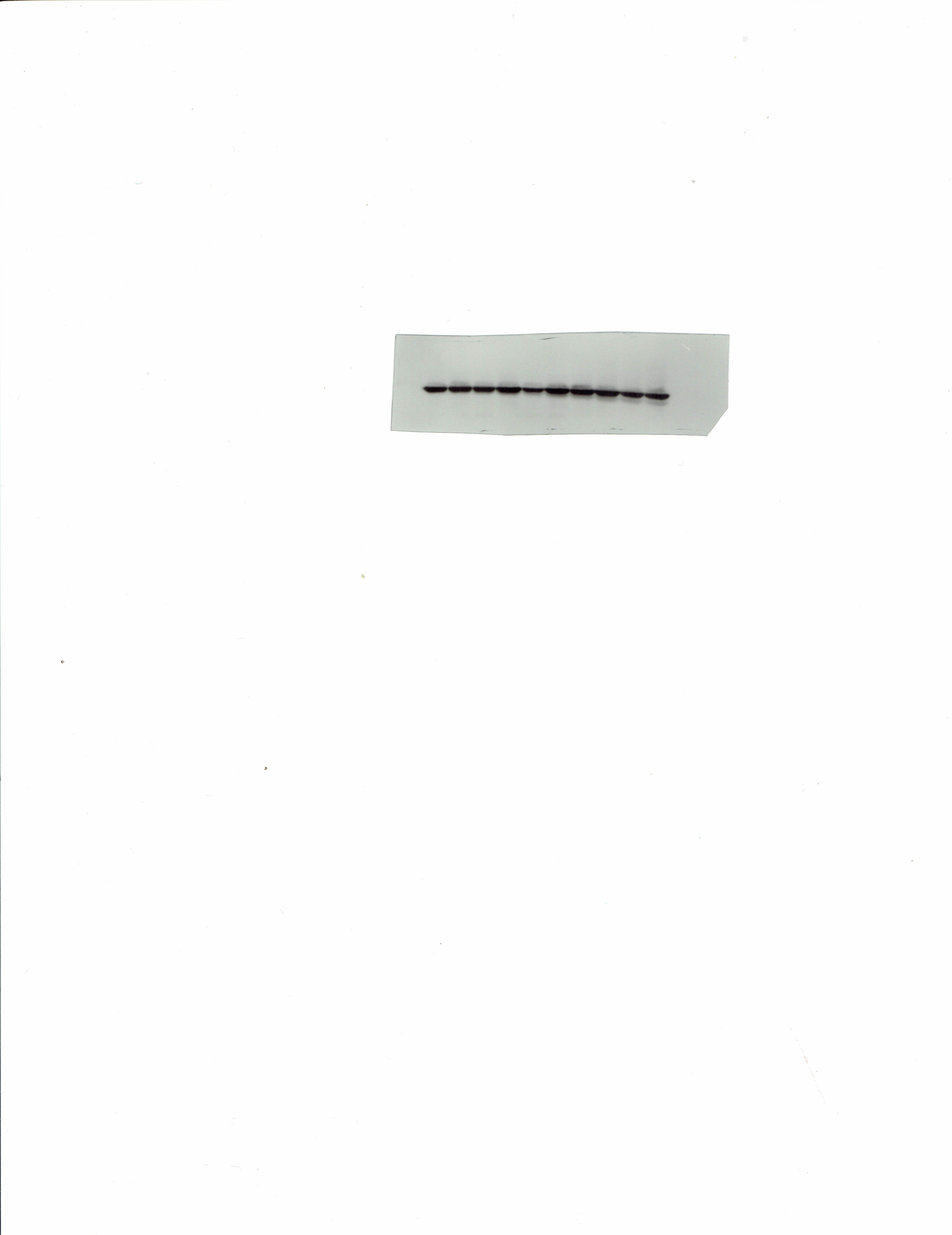

Supplement: Supplementary file 1 [file DataSheet2.ZIP › AO Neuro Manuscript_WB Figures/C1_Bactin.jpg]

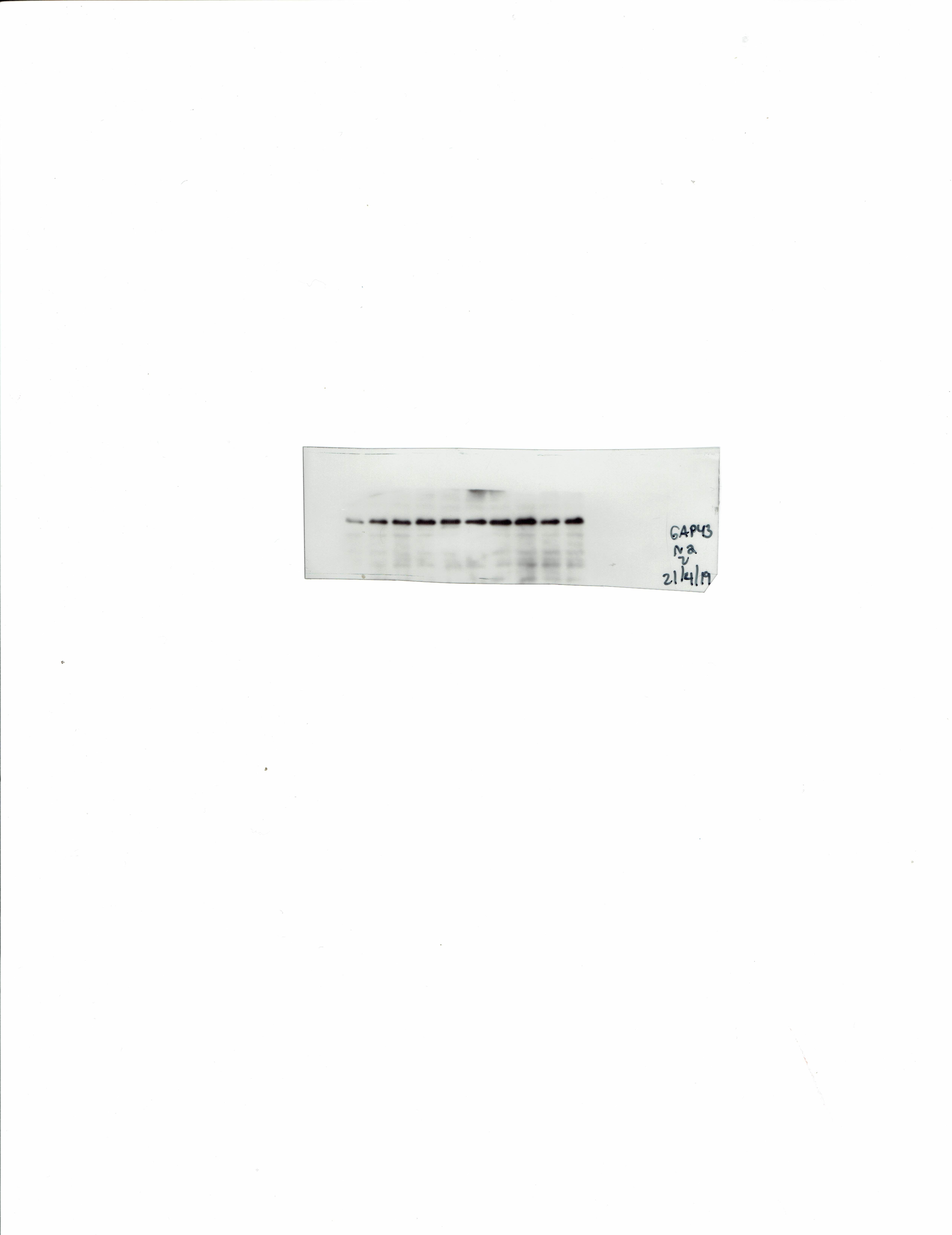

Supplement: Supplementary file 1 [file DataSheet2.ZIP › AO Neuro Manuscript_WB Figures/C1_GAP43.jpg]

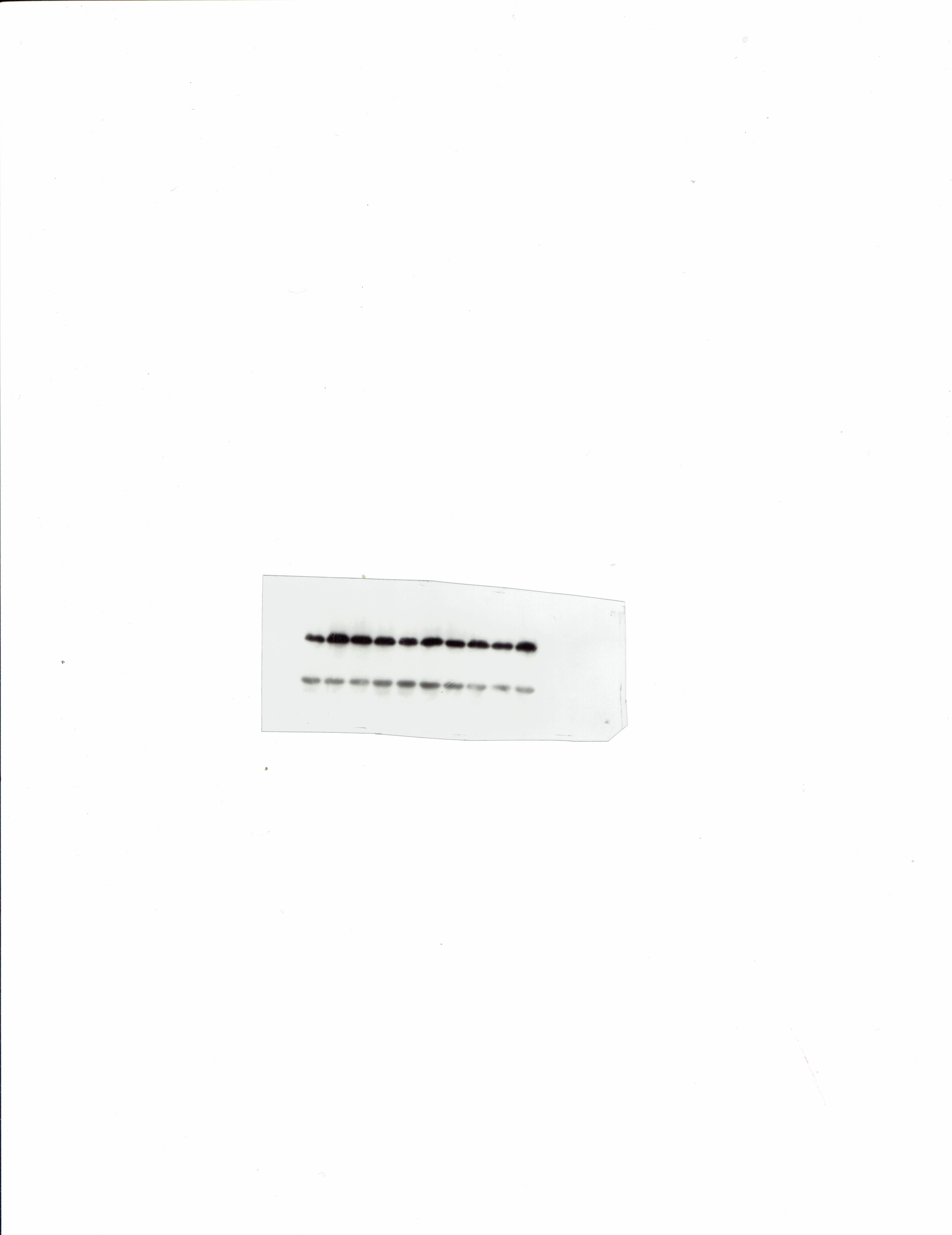

Supplement: Supplementary file 1 [file DataSheet2.ZIP › AO Neuro Manuscript_WB Figures/C2_GAP43_Bactin.jpg]

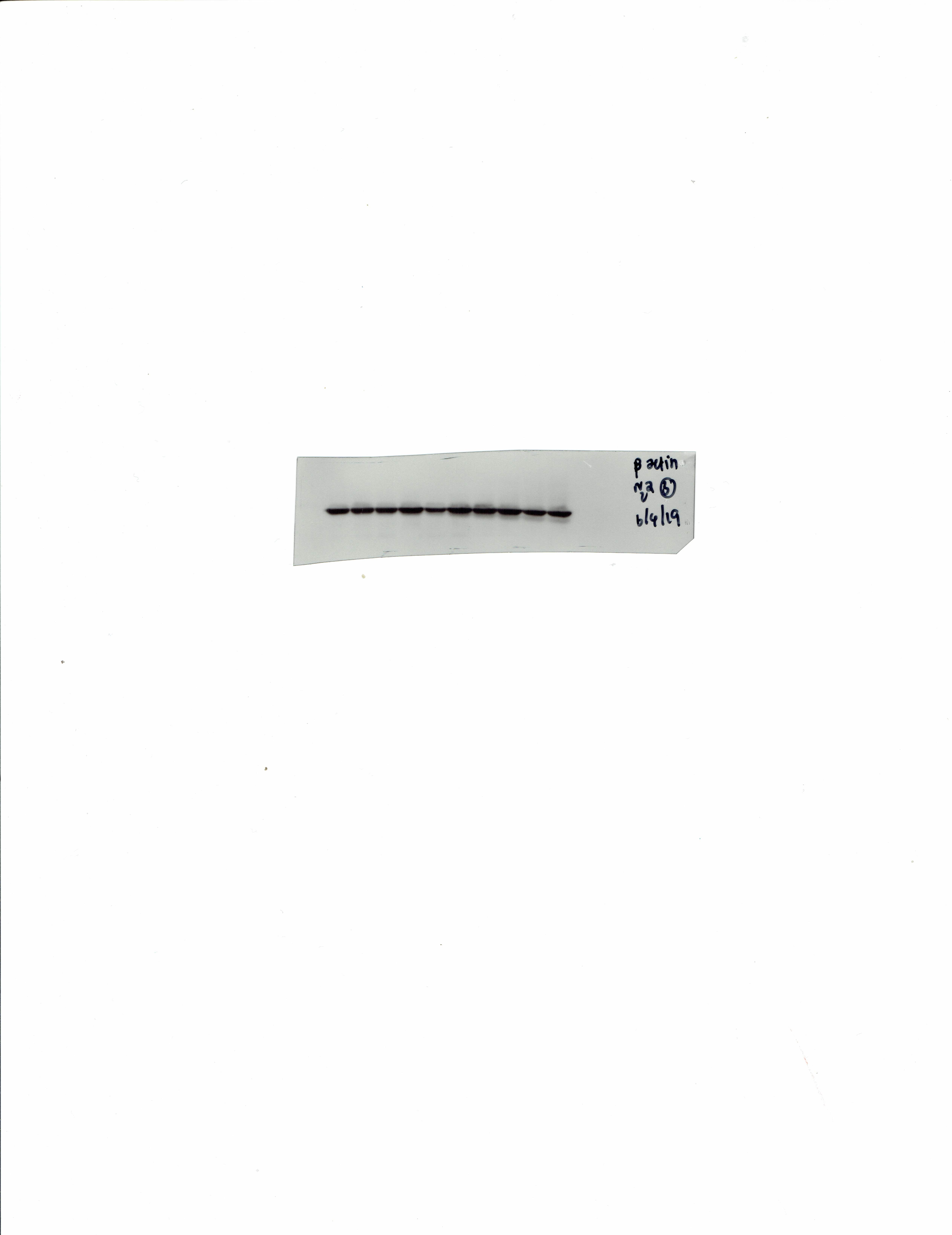

Supplement: Supplementary file 1 [file DataSheet2.ZIP › AO Neuro Manuscript_WB Figures/D1_Bactin.jpg]

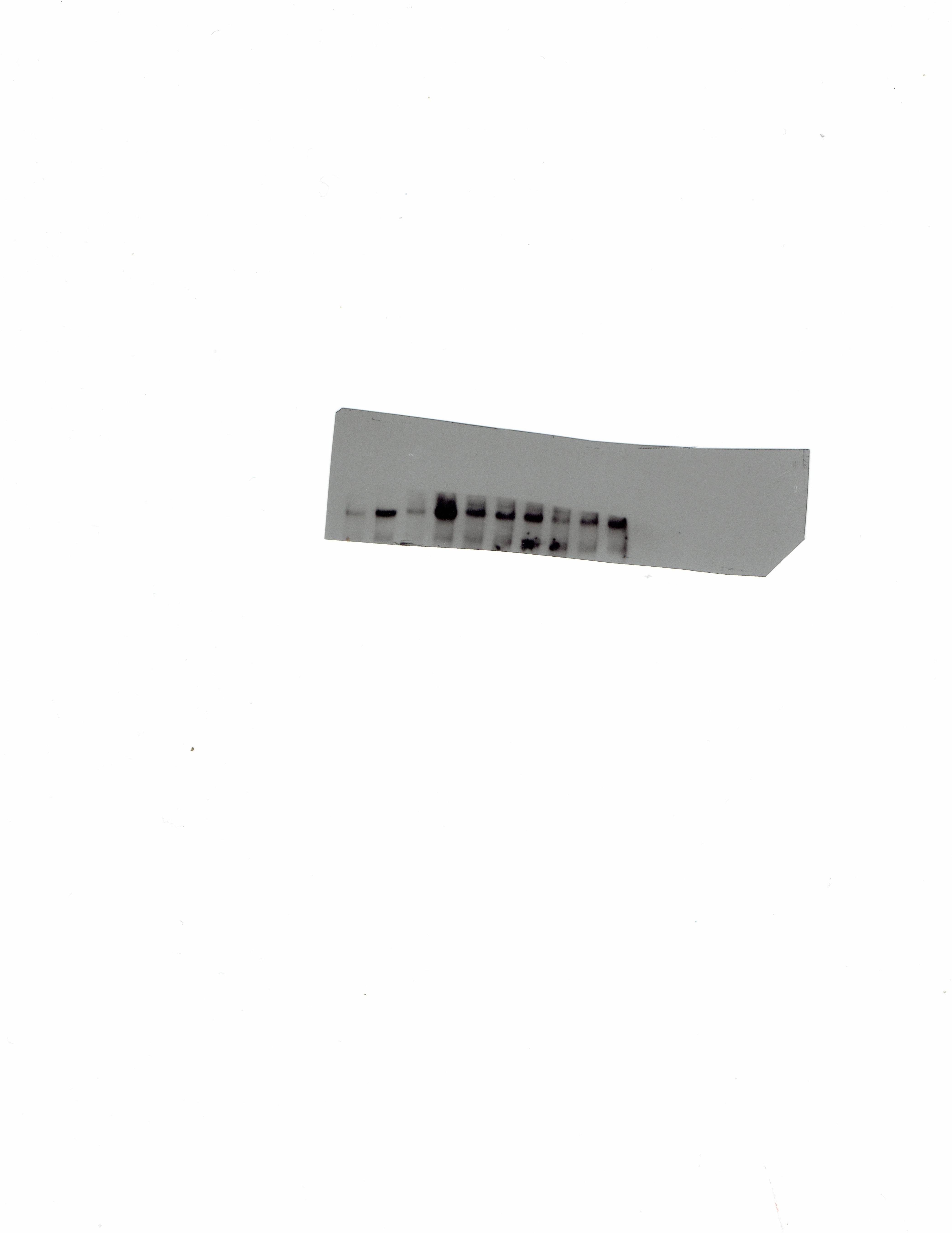

Supplement: Supplementary file 1 [file DataSheet2.ZIP › AO Neuro Manuscript_WB Figures/D1_Ten4.jpg]

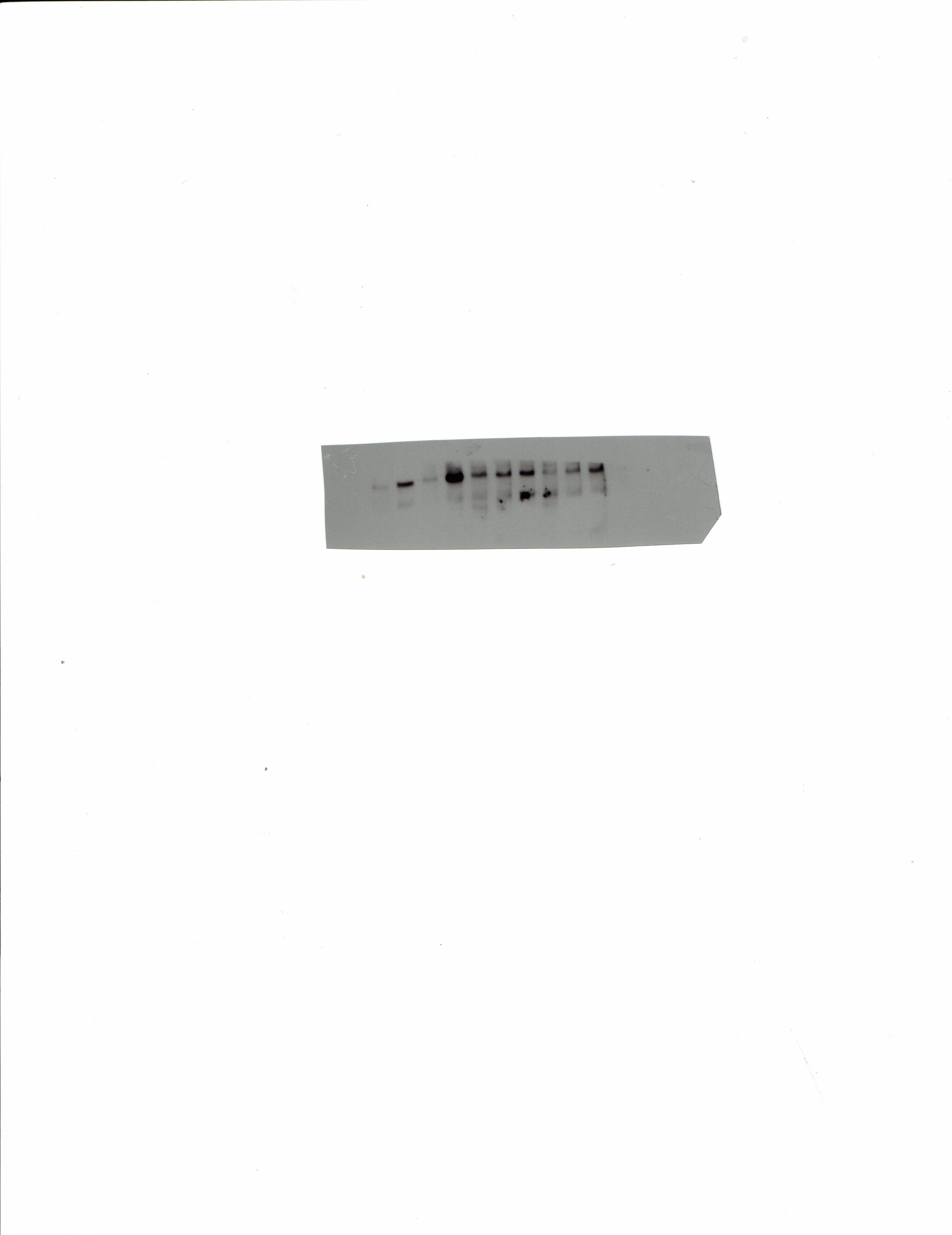

Supplement: Supplementary file 1 [file DataSheet2.ZIP › AO Neuro Manuscript_WB Figures/D2_Ten4.jpg]

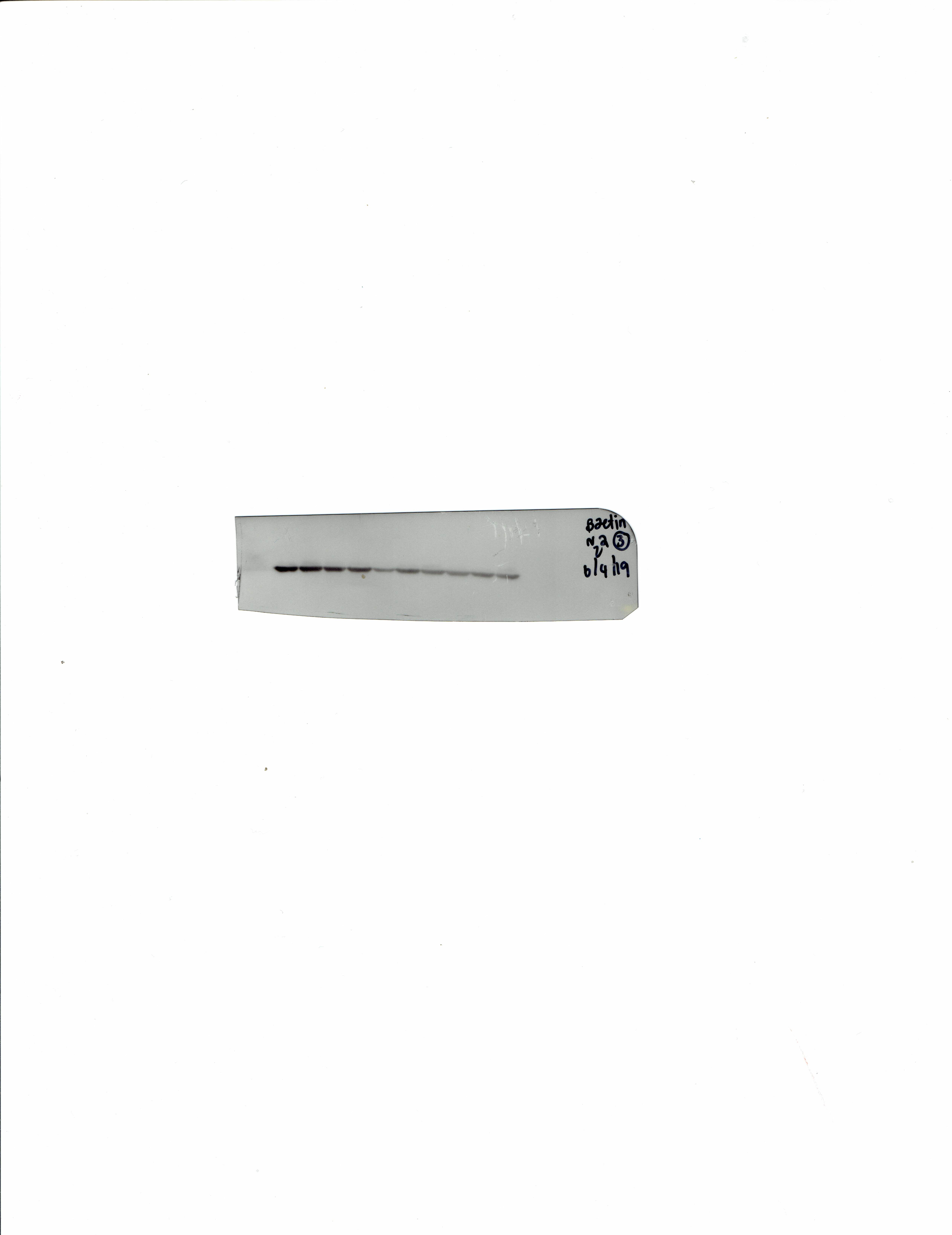

Supplement: Supplementary file 1 [file DataSheet2.ZIP › AO Neuro Manuscript_WB Figures/D3_Bactin.jpg]

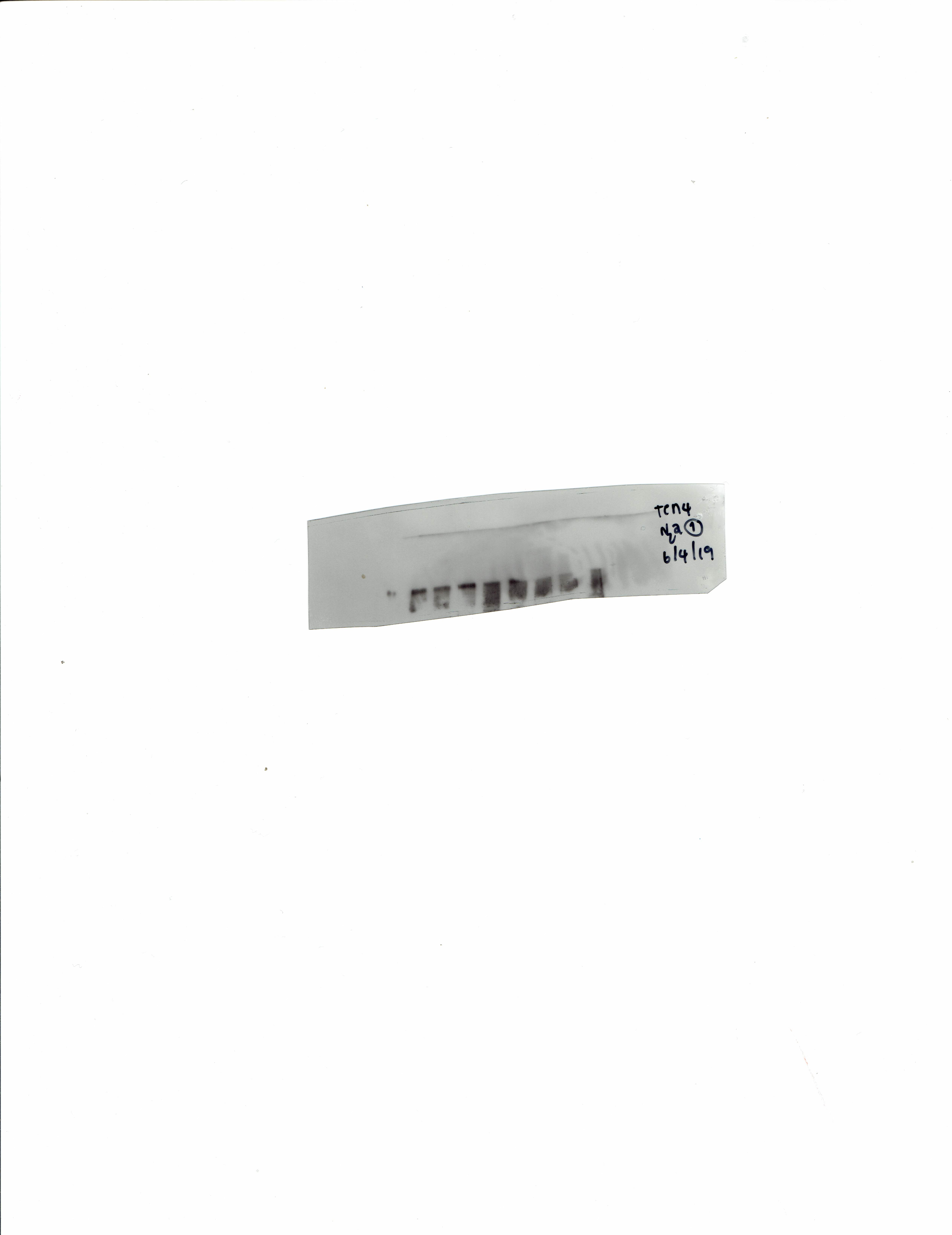

Supplement: Supplementary file 1 [file DataSheet2.ZIP › AO Neuro Manuscript_WB Figures/D3_Ten4.jpg]

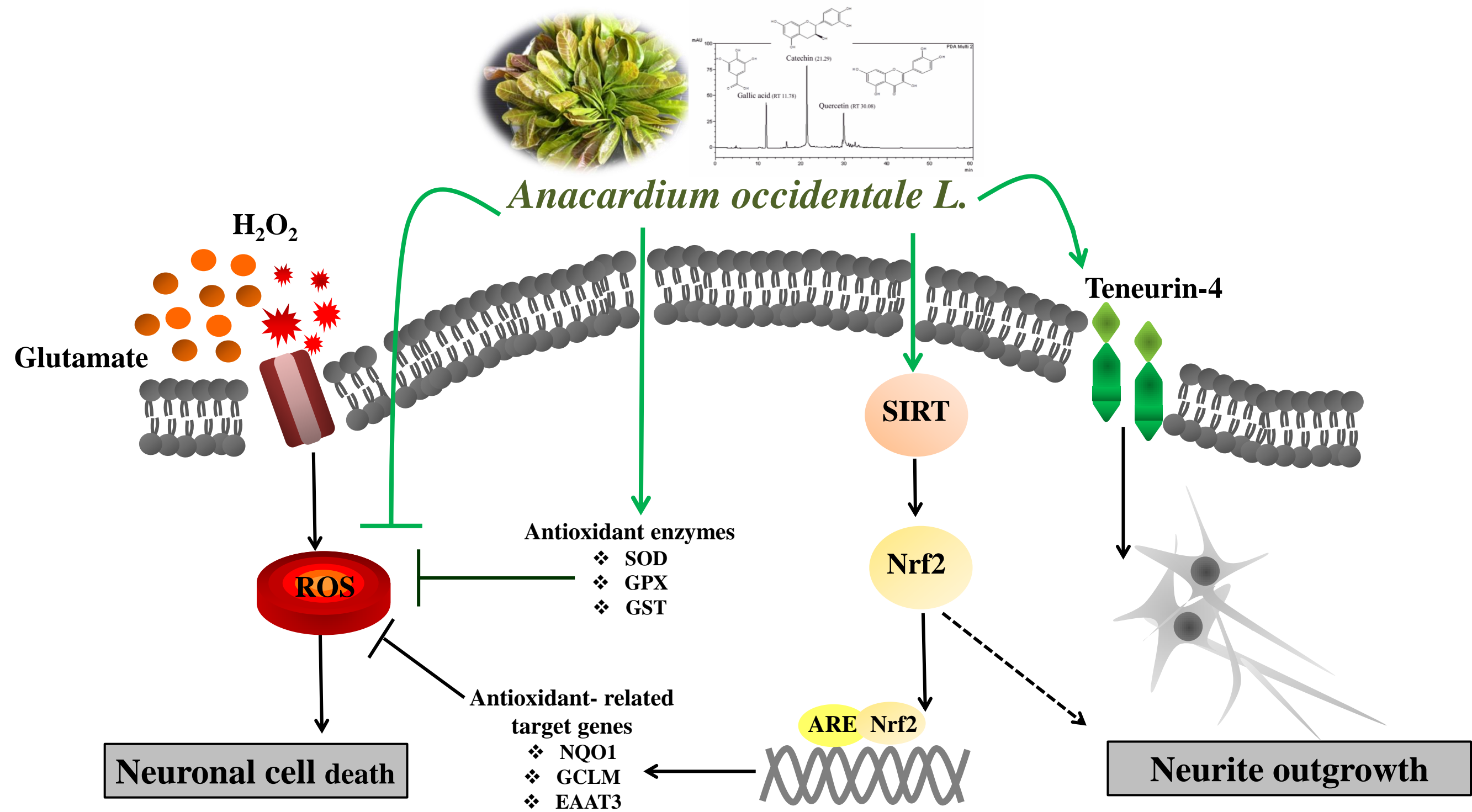

Supplement: Supplementary file 3 [file Image1.pdf]
