# Supplementary material for: Anacardium Occidentale L. Leaf Extracts Protect Against Glutamate/H2O2-Induced Oxidative Toxicity and Induce Neurite Outgrowth: The Involvement of SIRT1/Nrf2 Signaling Pathway and Teneurin 4 Transmembrane Protein
Source: Front Pharmacol. 2021 Apr 23;12:627738. doi: 10.3389/fphar.2021.627738 (PMC8114061; doi:10.3389/fphar.2021.627738)
Supplement: Supplementary file 2 [file DataSheet1.docx]

Supplementary Material

# Supplementary Materials and Methods

**1.1 Qualitative phytochemical screening**

For GLC-MS, chromatographic separation was carried out on a Clarus 600 GC-MS system (Perkin Elmer, Shelton, CT, USA) equipped with a 30 m × 0.25 mm × 0.25 μm Elite-5MS column (Perkin Elmer, USA). Oven temperature was set at 40 °C and increased by 5 °C/min until it reached 250 °C; carrier gas was helium at a constant flow of 1 mL/min. MS parameters: electron impact mode (EI) at ionization voltage of 70 eV, ion source temperature of 200 °C, and a scan range of 40–600 Da.

For HPLC, SHIMADZU LC-10 HPLC equipped with an analytical C18 reversed-phase column (ODS3 C18, 4.6 × 250 mm i.d., 5 µm particle size) and UV detector (best condition at 220 nm) was used. The mobile phase consisted of 0.02 M sodium acetate buffered to a pH of 4 with 0.0125 M citric acid containing 0.042 M methanesulfonic acid and 0.1 mM EDTA. Flow rate was set at 1 mL/min. Working standard solutions were freshly prepared in 0.05 M perchloric acid containing 0.1 mM Na2EDTA on ice and stored at −20 °C before using. Peaks were identified by comparing the retention time of each peak in the sample solution, where each individual peak was further compared to the standard solution of gallic acid, catechin, quercetin, octadecatrienolic acid (linolenic acid), and hexadecanoic (palmitic acid; Sigma-Aldrich, USA) served as an internal standard.

**1.2 Determination of cell viability**

Cell viability was evaluated by measuring MTT and LDH assay. To perform the MTT assay, after each treatment, the culture medium was added with 0.5 mg/mL MTT and incubated for 3 h at 37 °C. Then, all solution was removed, and the formazan crystals were solubilized by DMSO-ethanol mixture (1:1, v/v). The absorbance at 550 nm was measured using an EnSpire® Multimode Plate Reader (Perkin-Elmer, Waltham, MA, USA). Results were expressed as a percentage relative to the DMSO control.

To assess the LDH assay, the activity of LDH release in culture medium was measured using the CytoTox 96® assay (Promega) according to the manufacturer’s instructions. After each treatment, the culture supernatant was incubated with a substrate mix for 30 min in the dark at RT, followed by the addition of a stop solution. The absorbance at 490 nm was read using an EnSpire® Multimode Plate Reader (Perkin-Elmer, Waltham, MA, USA). Results were expressed as a percentage of maximum LDH release obtained by complete cell lysis.

**1.3 RNA isolation and quantitative RT-PCR**

The amount of RNA was determined by measuring absorbance at 260 nm. Then, 1 μg of total RNA was used for cDNA synthesis using AccuPower RT PreMix (Bioneer) and oligo (dT). All real-time PCR reactions were performed in an Exicycler™ 96 (Bioneer). PCR conditions: 95 °C for 15 min, followed by 45–55 cycles of denaturation at 95 °C for 15 s, and primer annealing/extension at 55 °C for 30 s. Melting-curve analysis was performed to determine primer specificity. The relative expression of each gene was normalized against the internal control gene (β-actin), and expression levels were analyzed using the 2-ΔΔCT method. The gene-specific sequences of the primers were SOD1 (forward: 5'- CAGGACCTCATTTTAATCCTCAC-3', reverse: 5'- CCCAGGTCTCCAACATGC-3'), CAT (forward: 5'- CAGCGACCAGATGAAGCA-3', reverse: 5'- CTCCGGTGGTCAGGACAT-3'), GPx (forward: 5'- ACAGTCCACCGTGTATGCCTTC-3', reverse: 5'- CTCTTCATTCTTGCCATTCTCCTG-3'), GSTo1 (forward: 5'- CAGCGATGTCGGGAGAAT-3', reverse: 5'- GGCAGAACCTCATGCTGTAGA-3'), GSTa2 (forward: 5'- TCTGACCCCTTTCCCTCTG-3', reverse: 5'- GCTGCCAGGATGTAGGAACT-3'), NQO1 (forward: 5’-CGACAACGGTCCTTTCCAGA-3′, reverse: 5’-TCCCAGACGGTTTCCAGAC-3′), GCLM (forward: 5’-GGAGCTTCGGGACTGTATCC-3′, reverse: 5’-AACTCCAAGGACGGAGCAT-3′), EAAT3 (forward: 5’-ATGATCTCGTCCAGTTCGGC-3′, reverse: 5’-TGACGATCTGCCC AATGCTT-3′) and β-actin (forward: 5'- GGCTGTATTCCCCTCCATCG-3', reverse: 5'- CCAGTTGGTAACAATGCCATGT-3') as normalization control.

**1.4 Measurement of neurite outgrowth and neurite-bearing cells**

Neuro-2a cells (15000 cells/well) were seeded in 6-well tissue culture plates in 10% FBS medium for 12-18 h. Media were carefully removed and washed with PBS. After that, the cells were treated with different concentrations of AOH (0.25-1 μg/mL) and AOM (0.5-10 μg/mL) in starving condition (1% FBS medium) for 48 h.

The Neuro-2a cells (100 cells /treatment) were randomly photographed using a bright-field microscope under 10 × magnifications. The cells were marked as differentiated if one or more neurites were longer than the diameter of the cell body. Neurite length was measured from the cell membrane of the body cell to the end of the growth cone and the percentage of neurite-bearing cells was quantified with ImageJ software (National Institutes of Health, Bethesda, MD). The expression of a specific neuronal differentiation marker, growth-associated protein 43 (GAP43), was investigated by real-time PCR and western blot analysis. The cells in complete growth medium (10% FBS) were used as negative control, and cells in starving condition (1% FBS) medium were used as the control. The cells were treated with 20 μM retinoic acid regarding the positive control.

#

Supplementary Figures

**Supplementary Figure 1.** Knockdown efficiency of Ten-4 by siRNA (siTen-4).

Ten-4 mRNA expression significantly decreased in siTen-4-Neuro-2a cells (62.26% compared to siCont-Neuro-2a cells). β-actin was used as the internal control for RT-PCR assay. All data were normalized to 10% FBS control levels in siCont-Neuro-2a cells and shown as the mean ± SEM in at least three independent experiments. *****p* < 0.0001 compared to the siCont-Neuro-2a cells in 1% FBS by one-way ANOVA following Bonferroni’s method (posthoc).





**Supplementary Figure 2.** The optimal concentrations of AO extracts for neurite outgrowth assays. Cell viability by treatment with different concentrations of AO hexane **(a)** and methanol **(b)** extracts for 48 h in Neuro-2a cells. Treatment of the Neuro-2a cells (15000 cells/well) with 0.25-1 µg/mL AO hexane extracts and 0.5-10 µg/mL AO methanol extracts for 48 h caused no significant changed in cell viability. However, treatment of cells with 10-50 µg/mL AO hexane extracts significantly reduced cell viability in a concentration-dependent manner. Average of neurite lengths **(ce**) and the percentage of neurite-bearing cells **(df)** after treatment with different concentrations of AO extracts. Treatment of the Neuro-2a cells (15000 cells/well) with 0.25-1 µg/mL AO hexane extracts and 0.5-10 µg/mL AO methanol extracts for 48 h significantly increased the average of neurite lengths **(ce)** and the percentage of neurite-bearing cells in a concentration-dependent manner. All data are shown as the mean ± SEM in at least three independent experiments. **p* < 0.05, ***p* < 0.01, ****p* < 0.001 and *****p* < 0.0001, compared to the untreated control or the 1% FBS control; #### *p* < 0.0001, compared to the 10% FBS control by one-way ANOVA following Bonferroni’s method (posthoc).


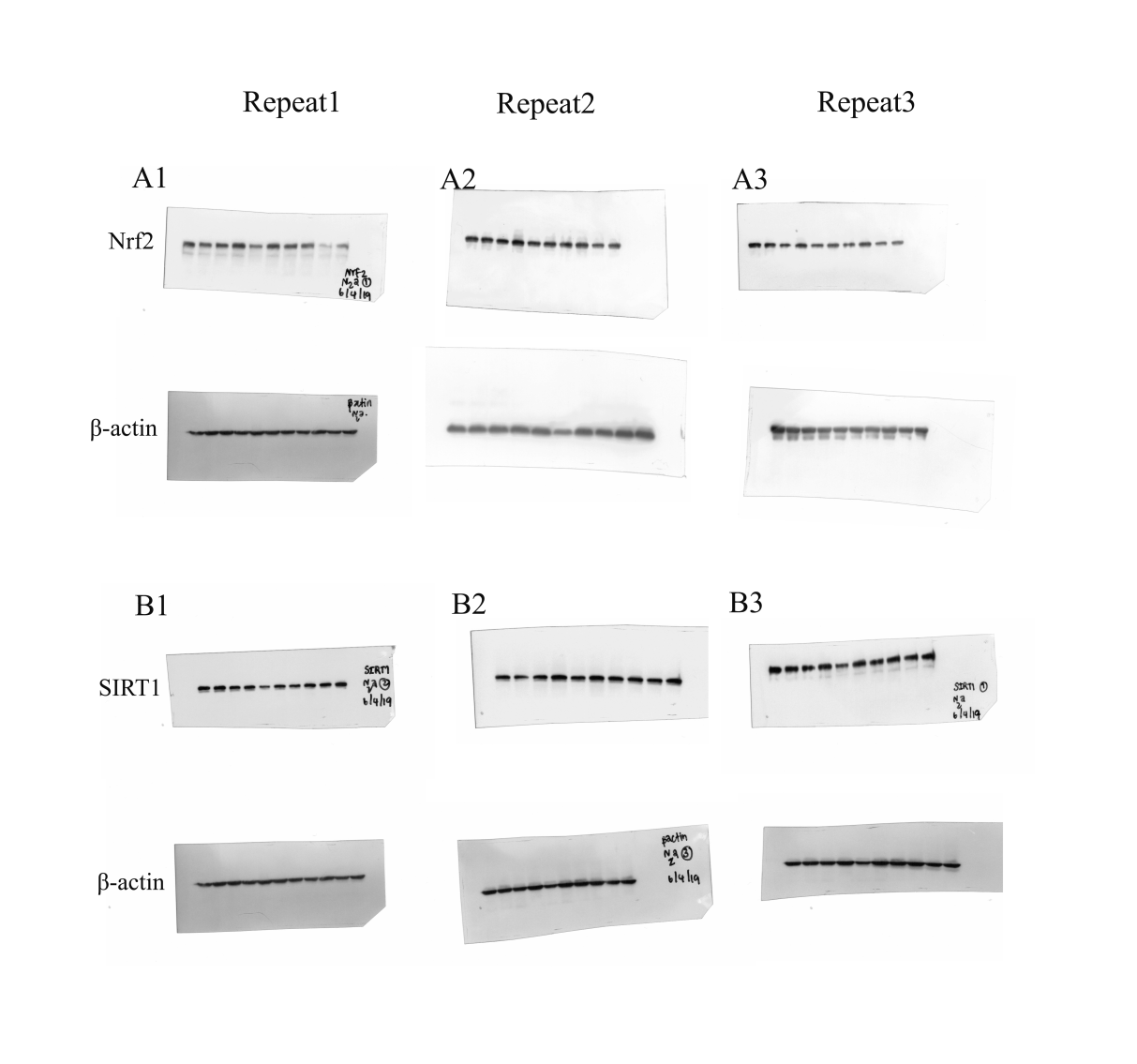


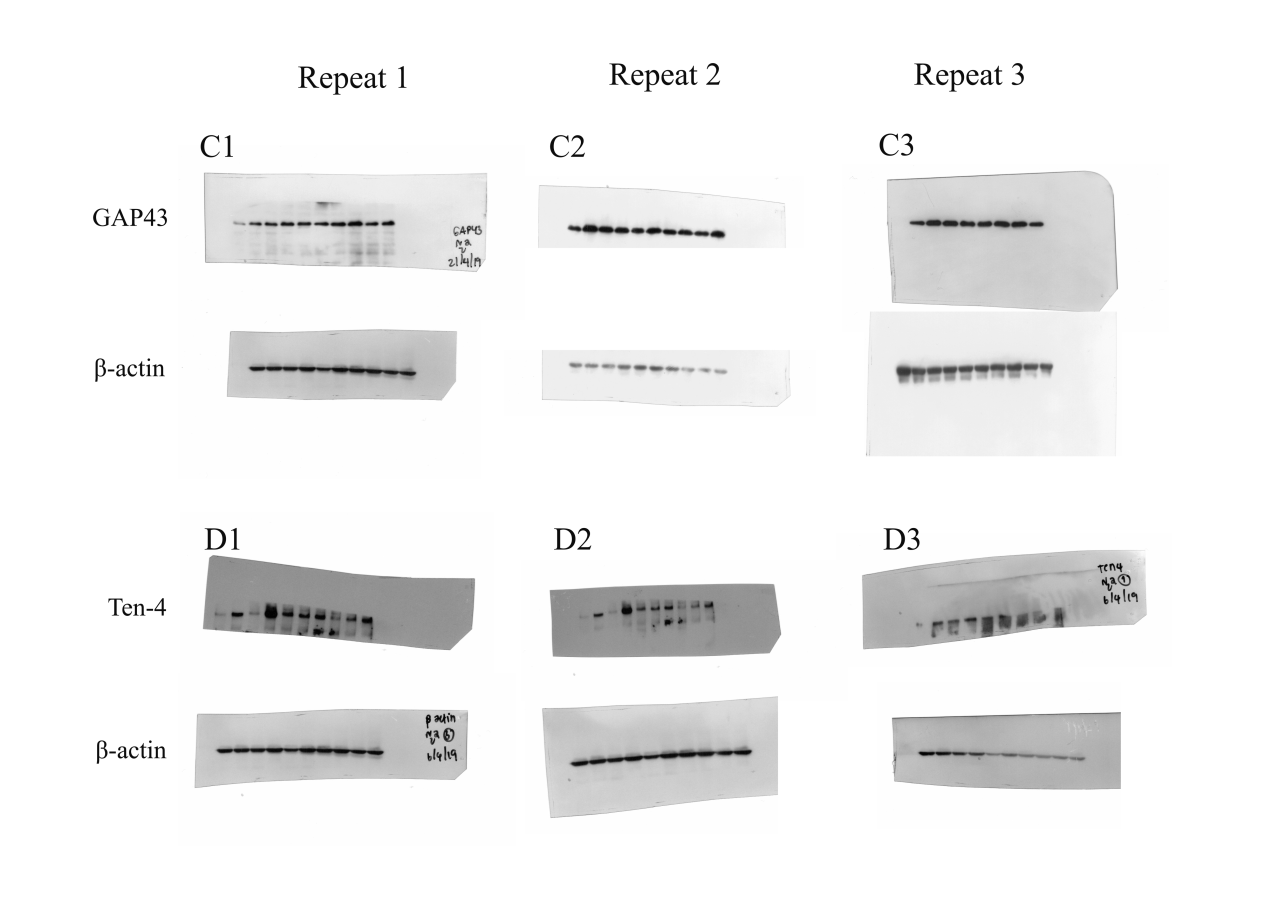


**Supplementary Figure 3.** The full images of electrophoretic blots by WB analysis.

Figure A1-A3, represent Nrf-2 and β-actin protein expressions in Neuro-2a cells (AOH:band5, AOM:band6, control:band9). Figure B1-B3, represent SIRT1 and β-actin protein expressions in Neuro-2a cells (AOH:band5, AOM:band6, control:band9). Figure C1-C3, represent GAP-43 and β-actin protein expressions in Neuro-2a cells (10%FBS:band1, 1%FBS:band2, AOH:band3, AOM:band4). Figure D1-D3, represent Ten-4 and β-actin protein expressions in Neuro-2a cells (1%FBS:band1, AOM:band2, AOH:band3). D2 and B2 were run used the same control (β-actin).


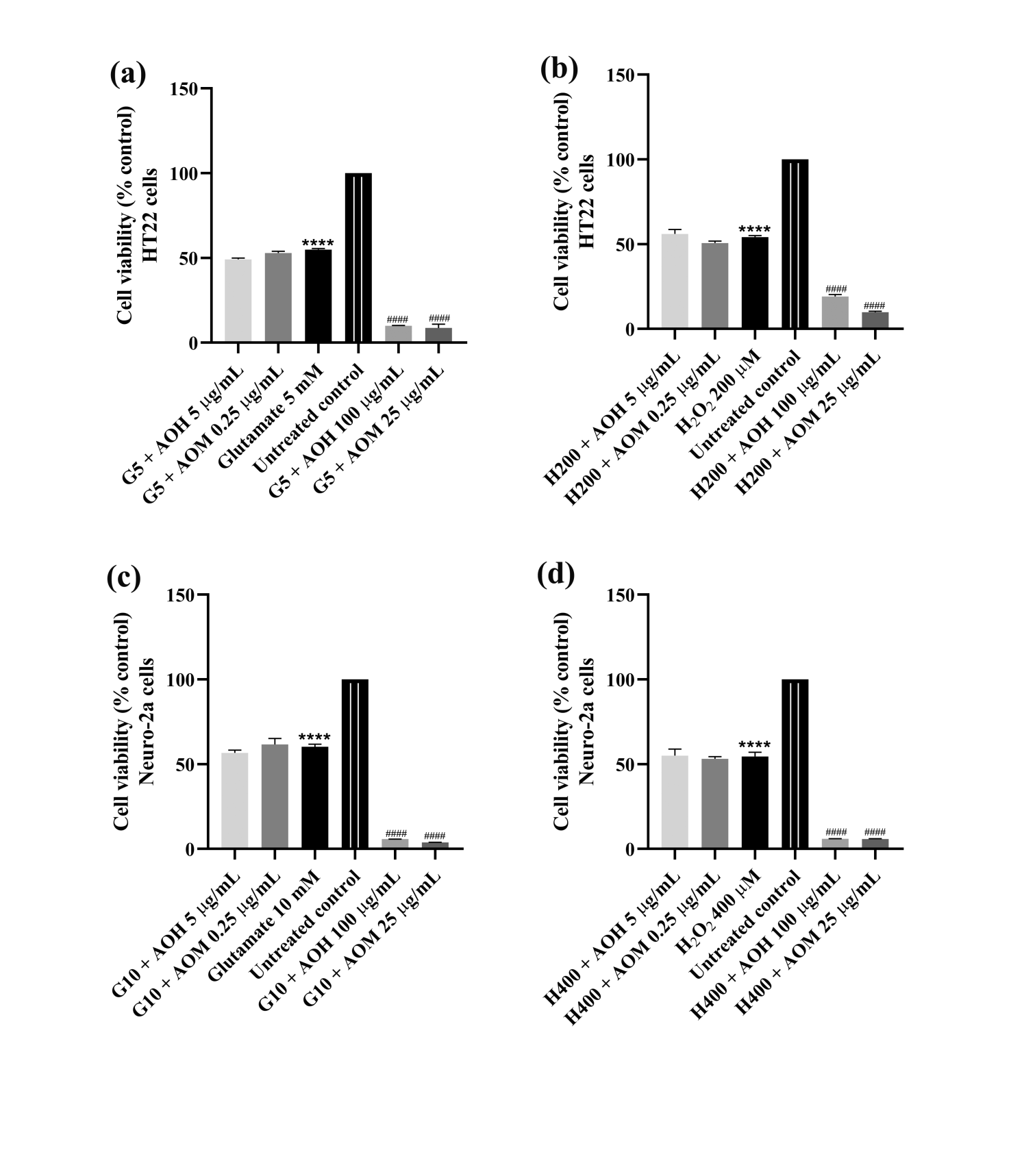


**Supplementary Figure 4. Effect of AO extracts on the survival rate and the intracellular ROS of HT22 cells and wild-type (N2) worms.**

AO extracts at low concentrations (5 µg/mL AOH and 0.25 µg/mL AOM) failed to protect against glutamate **(a,c)** and H_2_O_2_ **(b,d)** –induced neurotoxicity in both HT22 **(a,b)** and Neuro-2a cells **(c,d)**. Samples were treated with AO extracts for 48 h and exposed to glutamate (G5: 5 mM glutamate, G10: 10 mM glutamate) for 18 h (HT22 cells) or 24 h (Neuro-2a cells) to induce toxicity. The results are expressed as the means ± SEM of independent experiments (n=3). *****p* < 0.0001 compared to the untreated control; ^####^*p* < 0.0001 compared to the group exposed to glutamate only.

**
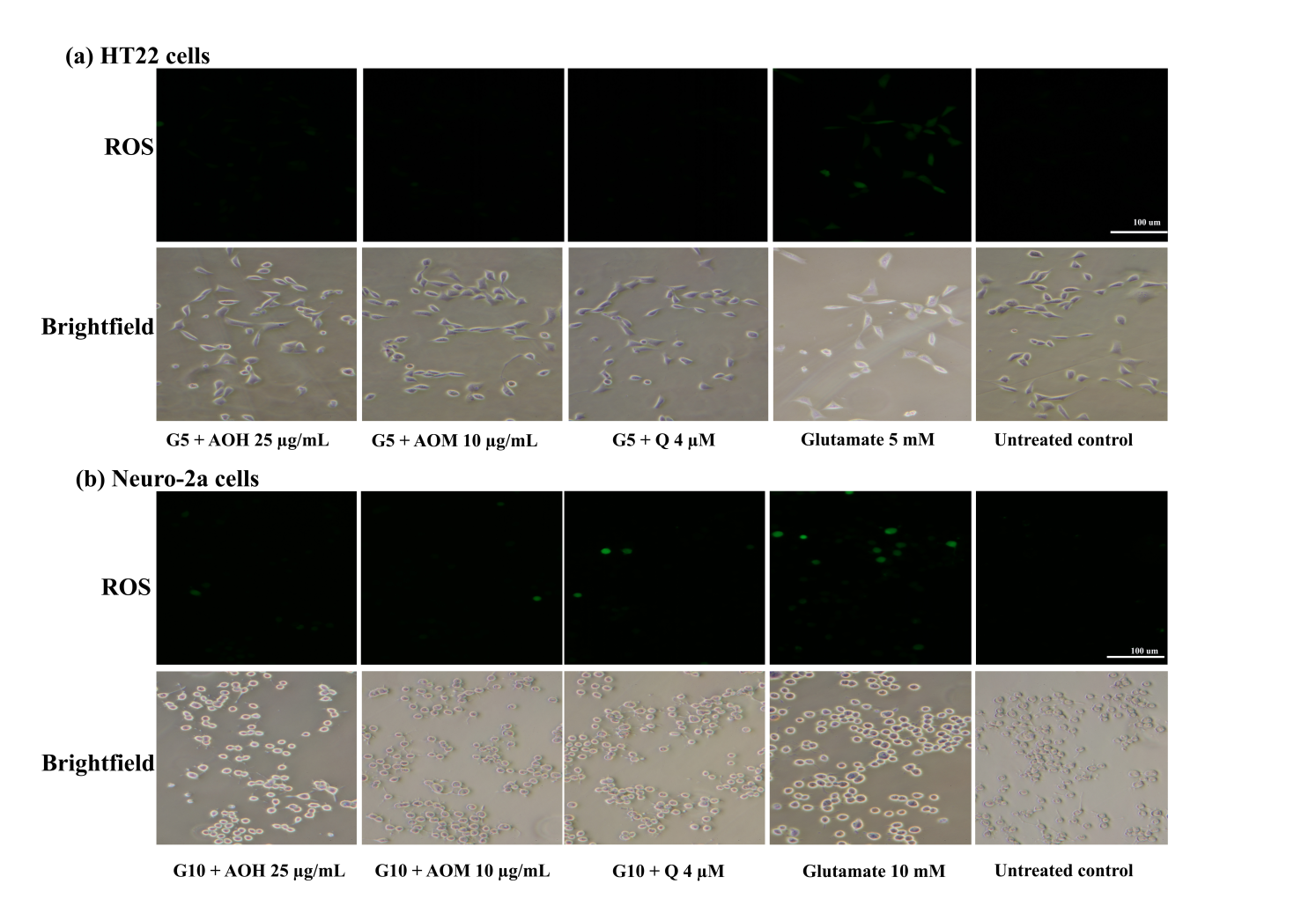
**

**Supplementary Figure 5.** Representative microscopy images of phase contrast and DCFH-DA staining of HTT2 and Neuro-2a cells after treated with AO extracts. Samples were treated with AO extracts for 48 h and exposed to glutamate (G5: 5 mM glutamate, G10: 10 mM glutamate) for 12 h (HT22 cells) or 18 h (Neuro-2a cells) to induce oxidative stress.

**
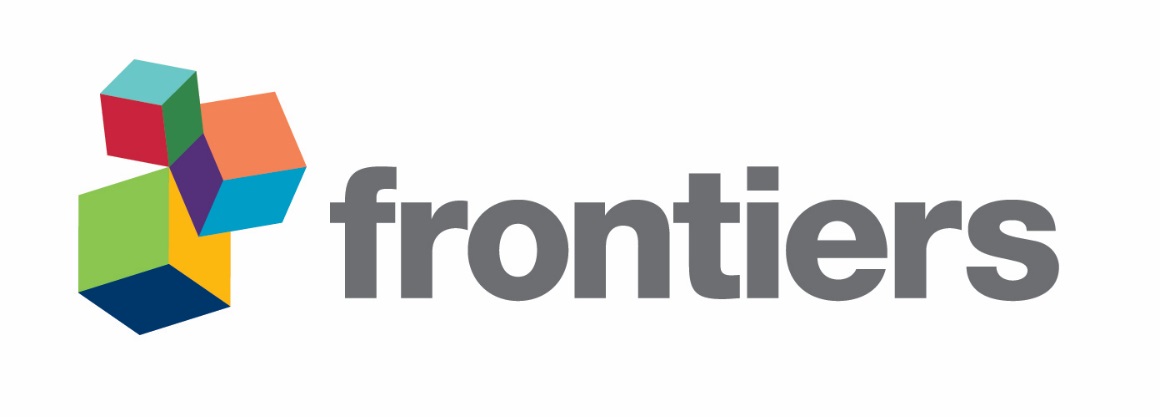
**
